# Global transcriptome response in *Lactobacillus sakei *during growth on ribose

**DOI:** 10.1186/1471-2180-11-145

**Published:** 2011-06-24

**Authors:** Anette McLeod, Lars Snipen, Kristine Naterstad, Lars Axelsson

**Affiliations:** 1 Nofima Mat AS, Norwegian Institute of Food, Fisheries and Aquaculture Research, Osloveien 1, Ås, NO-1430, Norway; 2 Department of Chemistry, Biotechnology and Food Science, Norwegian University of Life Sciences, P.O. Box 5003, Ås, NO-1432, Norway

## Abstract

**Background:**

*Lactobacillus sakei *is valuable in the fermentation of meat products and exhibits properties that allow for better preservation of meat and fish. On these substrates, glucose and ribose are the main carbon sources available for growth. We used a whole-genome microarray based on the genome sequence of *L. sakei *strain 23K to investigate the global transcriptome response of three *L. sakei *strains when grown on ribose compared with glucose.

**Results:**

The function of the common regulated genes was mostly related to carbohydrate metabolism and transport. Decreased transcription of genes encoding enzymes involved in glucose metabolism and the L-lactate dehydrogenase was observed, but most of the genes showing differential expression were up-regulated. Especially transcription of genes directly involved in ribose catabolism, the phosphoketolase pathway, and in alternative fates of pyruvate increased. Interestingly, the methylglyoxal synthase gene, which encodes an enzyme unique for *L. sakei *among lactobacilli, was up-regulated. Ribose catabolism seems closely linked with catabolism of nucleosides. The deoxyribonucleoside synthesis operon transcriptional regulator gene was strongly up-regulated, as well as two gene clusters involved in nucleoside catabolism. One of the clusters included a ribokinase gene. Moreover, *hprK *encoding the HPr kinase/phosphatase, which plays a major role in the regulation of carbon metabolism and sugar transport, was up-regulated, as were genes encoding the general PTS enzyme I and the mannose-specific enzyme II complex (EII^man^). Putative catabolite-responsive element (*cre*) sites were found in proximity to the promoter of several genes and operons affected by the change of carbon source. This could indicate regulation by a catabolite control protein A (CcpA)-mediated carbon catabolite repression (CCR) mechanism, possibly with the EII^man ^being indirectly involved.

**Conclusions:**

Our data shows that the ribose uptake and catabolic machinery in *L. sakei *is highly regulated at the transcription level. A global regulation mechanism seems to permit a fine tuning of the expression of enzymes that control efficient exploitation of available carbon sources.

## Background

The *Lactobacillus sakei *species belongs to the lactic acid bacteria (LAB), a group of Gram-positive organisms with a low G+C content which produce lactic acid as the main end product of carbohydrate fermentation. This trait has, throughout history, made LAB suitable for production of food. Acidification suppresses the growth and survival of undesirable spoilage bacteria and human pathogens. *L. sakei *is naturally associated with the meat and fish environment, and is important in the meat industry where it is used as starter culture for sausage fermentation [1,2]. The bacterium shows great potential as a protective culture and biopreservative to extend storage life and ensure microbial safety of meat and fish products [3-6]. The genome sequence of *L. sakei *strain 23K has revealed a metabolic repertoire which reflects the bacterium's adaption to meat products and the ability to flexibly use meat components [7]. Only a few carbohydrates are available in meat and fish, and *L. sakei *can utilize mainly glucose and ribose for growth, a utilization biased in favour of glucose [7-9]. The species has been observed as a transient member of the human gastrointestinal tract (GIT) [10,11], and ribose may be described as a commonly accessible carbon source in the gut environment [12]. Transit through the GIT of axenic mice gave mutant strains which grow faster on ribose compared with glucose [13].

Glucose is primarily transported and phosphorylated by the phosphoenolpyruvate (PEP)-dependent carbohydrate phosphotransferase system (PTS). A phosphorylation cascade is driven from PEP through the general components enzyme I (EI) and the histidine protein (HPr), then via the mannose-specific enzyme II complex (EII^man^) to the incoming sugar. Moreover, glucose is fermented through glycolysis leading to lactate [7,8,14]. Ribose transport and subsequent phosphorylation are induced by the ribose itself and mediated by a ribose transporter (RbsU), a D-ribose pyranase (RbsD), and a ribokinase (RbsK) encoded by *rbsUDK*, respectively. These genes form an operon with *rbsR *which encodes the local repressor RbsR [15,16]. The phosphoketolase pathway (PKP) is used for pentose fermentation ending with lactate and other end products [8,17]. *L. sakei *also has the ability to catabolize arginine, which is abundant in meat, and to catabolize the nucleosides inosine and adenine, a property which is uncommon among lactobacilli [7,18].

By proteomics, we recently identified proteins involved in ribose catabolism and the PKP to be over-expressed during growth on ribose compared with glucose, while several glycolytic enzymes were less expressed. Moreover, also enzymes involved in pyruvate- and glycerol/glycerolipid metabolism were over-expressed on ribose [19]. Bacteria often use carbon catabolite repression (CCR) in order to control hierarchical utilization of different carbon sources. In low G+C content Gram-positive bacteria, the dominant CCR pathway is mediated by the three main components: (1) catabolite control protein A (CcpA) transcriptional regulator; (2) the histidine protein (HPr); and (3) catabolite-responsive element (*cre*) DNA sites located in proximity to catabolic genes and operons, which are bound by CcpA [20-23]. The HPr protein has diverse regulatory functions in carbon metabolism depending on its phosphorylation state. In response to high throughput through glycolysis, the enzyme is phosphorylated at Ser46 by HPr kinase/phosphorylase (HPrK/P). This gives P-Ser-HPr which can bind to CcpA and convert it into its DNA-binding-competent conformation. However, when the concentration of glycolytic intermediates drop, the HPrK/P dephosphorylates P-Ser-HPr [20,22-24]. Under low glucose concentrations, HPr is phosphorylated by E1 of the PTS at His15 to give P-His-HPr, which has a catalytic function in the PTS and regulatory functions by phosphorylation of catabolic enzymes and transcriptional regulators with a PTS regulation domain (PRD). Several P-EIIBs also phosphorylate different types of non-PTS proteins and regulate their activities [20-22]. Evidence for regulatory processes resembling glucose repression was shown both during lactose utilization [25] and catabolism of arginine [26,27] in *L. sakei*. A *cre *site has been reported upstream of the *rbs *operon [28], thus CcpA could likely be acting on the *rbs *operon as well as other catabolic genes and operons in this bacterium.

In the present study, we use a microarray representing the *L. sakei *23K genome and an additional set of sequenced *L. sakei *genes, to investigate the global transcriptome response of three *L. sakei *strains when grown on ribose compared with glucose. Moreover, we predict the frequency of *cre *sites presumed to be involved in CCR in the *L. sakei *23K genome sequence. Our objective was to identify differentially expressed genes between growth on the two sugars, and to increase the understanding of how the primary metabolism is regulated.

## Methods

### Bacterial strains, media and growth conditions

*L. sakei *23K is a plasmid-cured sausage isolate [29], and its complete genome sequence has been published [7]. *L. sakei *LS 25 is a commercial starter culture strain for salami sausage [30]. *L. sakei *MF1053 originates from fermented fish (Norwegian "rakfisk") [9]. The strains were maintained at -80°C in MRS broth (Oxoid) supplemented with 20% glycerol. Growth experiments were performed in a defined medium for lactobacilli [31] supplemented with 0.5% glucose (DMLG) or 0.5% ribose + 0.02% glucose (DMLRg) as described previously [19]. Samples were extracted at three different days from independent DMLG and DMLRg cultures from each strain grown at 30°C to mid-exponential phase (OD_600 _= 0.5-0.6) for a total of three sample sets (parallels).

### Microarrays

The microarrays used have been described by Nyquist et al. [32], and a description is available at http://migale.jouy.inra.fr/sakei/Supplement.html/. 70-mer oligonucleotide probes representing the *L. sakei *strain 23K genome and an additional set of sequenced *L. sakei *genes were printed in three copies onto epoxy glass slides (Corning).

### RNA extraction

Total RNA extraction was performed using the RNeasy Protect Mini Prep Kit (Qiagen) as described by Rud et al. [33]. The concentration and purity of the total RNA was analysed using NanoDrop ND-1000 (NanoDrop Technologies), and the quality using Agilent 2100 Bioanalyzer (Agilent Technologies). Sample criteria for further use in the transcriptome analysis were A_260_/A_280 _ratio superior to 1.9 and 23S/16S RNA ratio superior to 1.6.

### cDNA synthesis, labeling, and hybridization

cDNA was synthesized and labeled with the Fairplay III Microarray Labeling Kit (Stratagene, Agilent Technologies) as described previously [34]. After labeling, unincorporated dyes were removed from the samples using the QIAQuick PCR purification kit (Qiagen). The following prehybridization, hybridization, washing, and drying of the arrays were performed in a Tecan HS 400 Pro hybridization station (Tecan) as described by Nyquist et al. [32]. For studying the carbon effects, samples from DMLG and DMLRg were co-hybridized for each of the three strains. Separate hybridizations were performed for each strain on all three biological parallels. In order to remove potential biases associated with labelling and subsequent scanning, a replicate hybridization was performed for each strain for one of the three parallels, where the Cy3 and Cy5 dyes (GE Healthcare) used during cDNA synthesis were swapped. The hybridized arrays were scanned at wavelengths 532 nm (Cy3) and 635 nm (Cy5) with a Tecan scanner LS (Tecan). GenePix Pro 6.0 (Molecular Devices) was used for image analysis, and spots were excluded based on slide or morphology abnormalities.

### Microarray data analysis

Downstream analysis was done by the Limma package http://www.bioconductor.org in the R computing environment http://www.r-project.org. Pre-processing and normalization followed a standard procedure using methods described by Smyth & Speed [35], and testing for differential expressed genes were done by using a linear mixed model as described by Smyth [36]. A mixed-model approach was chosen to adequately describe between-array variation and still utilize probe-replicates (three replicates of each probe in each array). An empirical Bayes smoothing of gene-wise variances was conducted according to Smyth et al. [37], and for each gene the p-value was adjusted to control the false discovery rate (FDR), hence all p-values displayed are FDR-adjusted (often referred to as q-values in the literature).

### Validation of microarray data by qRT-PCR analysis

The microarray results were validated on selected regulated genes for the LS 25 strain by quantitative real-time reverse transcriptase PCR (qRT-PCR) performed as described previously [38]. Primers and probes (Additional file 1, Table S3) were designed using Primer Express 3.0 (Applied Biosystems). Relative gene expression was calculated by the Δ*C_T _*method, using the DNA gyrase subunit alpha gene (*gyrA*) as the endogenous reference gene.

### Microarray accession numbers

The microarray data have been deposited in the Array Express database http://www.ebi.ac.uk/arrayexpress/ under the accession numbers A-MEXP-1166 (array design) and E-MEXP-2892 (experiment).

### Sequence analysis

A prediction of *cre *sites in the *L. sakei *23K genome sequence (GeneBank acc. no. CR936503.1), both strands, was performed based on the consensus sequence TGWNAN**CG**NTNWCA (W = A/T, N = A/T/G/C), confirmed in Gram-positive bacteria [39]. We made a search with the consensus sequence described by the regular expression T-G-[AT]-X-A-X-C-G-X-T-X-[AT]-C-A, allowing up to two mismatches in the conserved positions except for the two center position, highlighted in boldface. All computations were done in R http://www.r-project.org.

## Results and Discussion

### Selection of *L. sakei *strains and growth conditions

We have previously investigated *L. sakei *strain variation [9], and used proteomics to study the bacterium's primary metabolism [19], providing us with a basis for choosing strains with interesting differences for further studies. The starter culture strain LS 25 showed the fastest growth rates in a variety of media, and together with strain MF1053 from fish, it fermented the highest number of carbohydrates [9]. The LS 25 strain belongs to the *L. sakei *subsp. *sakei*, whereas the 23K and MF1053 strains belong to *L. sakei *subsp. *carnosus *[9,19]. By identification of differentially expressed proteins caused by the change of carbon source from glucose to ribose, LS 25 seemed to down-regulate the glycolytic pathway more efficiently than other strains during growth on ribose [19]. For these reasons, LS 25 and MF1053 were chosen in addition to 23K for which the microarray is based on. Nyquist et al. [32] recently investigated the genomes of various *L. sakei *strains compared to the sequenced strain 23K by comparative genome hybridization (CGH) using the same microarray as in the present study. A large part of the 23K genes belongs to a common gene pool invariant in the species, and the status for each gene on the array is known for all the three strains [32].

As glucose is the preferred sugar, *L. sakei *grows faster when glucose is utilized as the sole carbon source compared with ribose [8,9,15]. However, glucose stimulates ribose uptake and a possible co-metabolism of these two sugars present in meat and fish has been suggested, a possibility that give the organism an advantage in competition with other microbiota [15,16,40]. To obtain comparable 2-DE gels between samples issued from bacteria grown on the two carbohydrates in our recent proteomic analysis, growth on ribose was enhanced by adding small amounts of glucose [19]. For the present transcriptome analysis we therefore chose the same growth conditions.

### Global gene expression patterns

**Figure 1:**
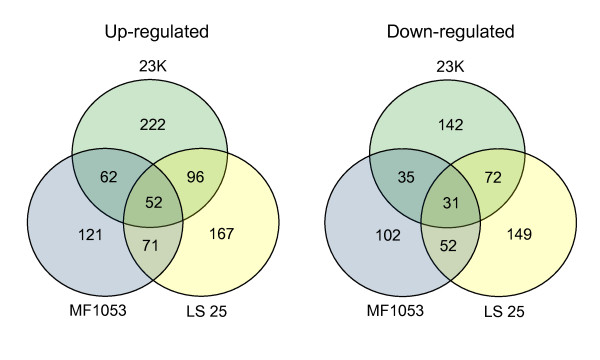
**Venn diagram showing the number of unique and common up- and down-regulated genes in *L. sakei *strains 23K, MF1053 and LS 25 when grown on ribose compared with glucose**.

A microarray representing the *L. sakei *23K genome and an additional set of sequenced *L. sakei *genes was used for studying the effect of carbon source on the transcriptome of *L. sakei *strains 23K, MF1053 and LS 25. Genes displaying a significant differential expression with a log_2 _ratio > 0.5 or < -0.5 were classified into functional categories according to the *L. sakei *23K genome database http://migale.jouy.inra.fr/sakei/genome-server and are listed in Table 1. The 23K strain showed differential expression for 364 genes within these limits, MF1053 and LS 25 for 223 and 316 genes, respectively. Among these, 88, 47 and 82, respectively, were genes belonging to the category of genes of 'unknown' function. Eighty three genes, the expression of which varied depending on the carbon source, were common to the three strains, among which 52 were up-regulated and 31 down-regulated during growth on ribose (Figure 1). The function of these common regulated genes was mostly related to carbohydrate transport and metabolism (34 genes, Table 1). The reliability of the microarray results was assessed by qRT-PCR analysis using selected regulated genes in the LS 25 strain. As shown in Table S4 in the additional material (Additional file 1), the qRT-PCR results were in agreement with the data obtained by the microarrays.

**Table 1:** Genes with significant differential expression in three *L. sakei *strains grown on ribose compared with glucose, FDR adjusted p-value less than 0.01 and log_2 _of > 0.5 or < -0.5 (log_2 _values > 1.0 or < -1.0 are shown in bold).

Gene locus	Gene	Description	23K	MF1053	LS 25
**Carbohydrate transport and metabolism**					
					
**Transport/binding of carbohydrates**					
LSA0185*	*galP*	Galactose:cation symporter	**1.2**		**1.7**
LSA0200*	*rbsU*	Ribose transport protein	**2.8**	**3.5**	**4.3**
LSA0353*	*lsa0353*	Putative cellobiose-specific PTS, enzyme IIB	**3.6**	**1.3**	**2.5**
LSA0449*	*manL*	Mannose-specific PTS, enzyme IIAB	**2.1**	**2.5**	**1.5**
LSA0450*	*manN*	Mannose-specific PTS, enzyme IIC	**1.9**	**2.0**	**1.4**
LSA0451*	*manM*	Mannose-specific PTS, enzyme IID	**2.4**	**1.0**	**2.1**
LSA0651*	*glpF*	Glycerol uptake facilitator protein, MIP family	**3.4**	**4.7**	**3.4**
LSA1050*	*fruA*	Fructose-specific PTS, enzyme IIABC			0.9
LSA1204*	*lsa1204*	Putative sugar transporter		**1.1**	
LSA1457*	*lsa1457*	Putative cellobiose-specific PTS, enzyme IIC		**2.3**	
LSA1462*	*ptsI*	PTS, enzyme I	0.8	**1.7**	0.9
LSA1463*	*ptsH*	Phosphocarrier protein HPr (histidine protein)		**1.2**	0.9
LSA1533	*lsa1533*	Putative cellobiose-specific PTS, enzyme IIA		**2.5**	**2.1**
LSA1690	*lsa1690*	Putative cellobiose-specific PTS, enzyme IIC	0.9		
LSA1792*	*scrA*	Sucrose-specific PTS, enzyme IIBCA	0.8		**1.1**
**Metabolism of carbohydrates and related molecules**					
LSA0123*	*lsa0123*	Putative sugar kinase, ROK family	**1.2**		
LSA0198	*ack1*	Acetate kinase (acetokinase)	**1.7**		**1.3**
LSA0254*	*lsa0254*	Putative carbohydrate kinase	**2.4**	0.8	**1.8**
LSA0292*	*budC*	Acetoin reductase (acetoin dehydrogenase) (meso-2,3-butanediol dehydrogenase)	**3.4**	**2.3**	**3.4**
LSA0444	*lsa0444*	Putative malate dehydrogenase	**3.4**	D	**2.1**
LSA0516	*hprK*	Hpr kinase/phosphorylase	**2.0**	**1.6**	**1.2**
LSA0664*	*loxL1N*	L-lactate oxidase (N-terminal fragment), degenerate	**1.2**		0.7
LSA0665*	*loxLI*	L-lactate oxidase (central fragment), degenerate	**1.0**		
LSA0666*	*loxL1C*	L-lactate oxidase (C-terminal fragment), degenerate	**1.0**		
LSA0974*	*pflB*	Formate C-acetyltransferase (pyruvate formate-lyase) (formate acetyltransferase)	**4.0**		
LSA0981	*aldB*	Acetolactate decarboxylase (alpha-acetolactate decarboxylase)		0.6	**1.9**
LSA0982	*als*	Acetolactate synthase (alpha-acetolactate synthase)			**1.9**
LSA0983	*lsa0983*	Putative aldose-1 epimerase	0.6		
LSA1032	*pyk*	Pyruvate kinase		-0.7	
LSA1080	*lsa1080*	Myo-inositol monophosphatase	0.6		0.8
LSA1082	*pdhD*	Pyruvate dehydrogenase complex, E3 component, dihydrolipoamide dehydrogenase	**2.8**	**2.5**	**2.1**
LSA1083	*pdhC*	Puruvate dehydrogenase complex, E2 component, dihydrolipoamide acetyltransferase	**3.4**	**3.7**	**2.7**
LSA1084	*pdhB*	Pyruvate dehydrogenase complex, E1 component, beta subunit	**3.2**	**3.3**	**2.2**
LSA1085	*pdhA*	Pyruvate dehydrogenase complex, E1 component, alpha subunit	**2.9**	**3.5**	**2.4**
LSA1141*	*ppdK*	Pyruvate phosphate dikinase	**1.0**		0.9
LSA1188*	*pox1*	Pyruvate oxidase	**2.3**	**3.1**	**2.1**
LSA1298	*ack2*	Acetate kinase (acetokinase)	**1.1**	0.9	0.9
LSA1343*	*eutD*	Phosphate acetyltransferase (phosphotransacetylase)	**2.0**	**1.0**	**1.6**
LSA1381	*lsa1381*	Putative acylphosphatase	-0.6	-0.5	
LSA1399*	*loxL2*	L-lactate oxidase	**3.4**	U	
LSA1630	*lsa1630*	Putative sugar kinase, ROK family	-0.6		-0.6
LSA1640*	*nanA*	N-acetylneuraminate lyase	**2.0**		D
LSA1641*	*nanE*	N-acylglucosamine/mannosamine-6-phosphate 2-epimerase	0.9		D
LSA1643*	*lsa1643*	Putative sugar kinase, ROK family	**1.8**		
LSA1668	*ack3*	Acetate kinase (acetokinase)	-0.7		**-1.1**
LSA1830*	*pox2*	Pyruvate oxidase	0.7		
**Intermediary metabolism**					
LSA0255*	*lsa0255*	Putative phosphoribosyl isomerase	**2.0**	**1.0**	**1.6**
**Specific carbohydrate metabolic pathway**					
LSA0201*	*rbsD*	D-ribose pyranase	**2.5**	**2.5**	**3.4**
LSA0202*	*rbsK*	Ribokinase	**3.0**	**3.9**	**4.3**
LSA0289*	*xpk*	Xylulose-5-phosphate phosphoketolase	**3.2**	**2.3**	**2.6**
LSA0297	*gntZ*	6-phosphogluconate dehydrogenase	**-1.2**	-0.9	**-1.7**
LSA0298	*gntK*	Gluconokinase	-0.8		
LSA0381	*zwf*	Glucose-6-phosphate 1-dehydrogenase	-0.6	-0.6	-0.6
LSA0649*	*glpK*	Glycerol kinase	**3.4**	**4.8**	**2.1**
LSA0650*	*glpD*	Glycerol-3-phosphate dehydrogenase	**2.3**	**2.2**	**2.0**
LSA0764*	*galK*	Galactokinase	**1.1**	0.7	**1.8**
LSA0765*	*galE1*	UDP-glucose 4-epimerase			**1.2**
LSA0766*	*galT*	Galactose-1-phosphate uridylyltransferase	**1.2**	0.8	**2.0**
LSA0767*	*galM*	Aldose 1-epimerase (mutarotase)	**1.3**		**2.0**
LSA1146*	*manA*	Mannose-6-phosphate isomerase	**1.4**	**1.3**	**1.5**
LSA1531	*lsa1531*	Putative beta-glucosidase		0.7	0.9
LSA1588	*nagA*	N-acetylglucosamine-6-phosphate deacetylase	0.6		
LSA1685	*rpiA*	Ribose 5-phosphate epimerase (ribose 5-phosphate isomerase)		**1.1**	0.8
LSA1710*	*lacM*	Beta-galactosidase, small subunit (lactase, small subunit)	**3.3**		**1.2**
LSA1711*	*lacL*	Beta-galactosidase, large subunit (lactase, large subunit)	**3.0**	**1.5**	**1.7**
LSA1790*	*scrK*	Fructokinase		**1.0**	**1.1**
LSA1791*	*dexB*	Glucan 1,6-alpha-glucosidase (dextran glucosidase)			**1.1**
LSA1795	*melA*	Alpha-galactosidase (melibiase)			-0.6
**Glycolytic pathway**					
LSA0131	*gpm2*	Phosphoglycerate mutase		0.7	
LSA0206	*gpm3*	Phosphoglycerate mutase	-0.7	-0.8	-0.9
LSA0609*	*gloAC*	Lactoylglutathione lyase (C-terminal fragment), authentic frameshift	**1.1**		0.7
LSA0803	*gpm4*	Phosphoglycerate mutase	0.5		0.5
LSA1033	*pfk*	6-phosphofructokinase	-0.6	**-1.1**	-0.5
LSA1157	*mgsA*	Methylglyoxal synthase	**2.3**	**1.4**	**1.7**
LSA1179	*pgi*	Glucose-6-phosphate isomerase	0.5		
LSA1527	*fba*	Fructose-bisphosphate aldolase	**-1.0**	-0.7	**-1.1**
LSA1606	*ldhL*	L-lactate dehydrogenase	**-1.0**	-0.9	**-1.5**
					
**Nucleotide transport and metabolism**					
**Transport/binding of nucleosides, nucleotides, purines and pyrimidines**					
LSA0013	*lsa0013*	Putative nucleobase:cation symporter	-0.9		**-1.5**
LSA0055	*lsa0055*	Putative thiamine/thiamine precursor:cation symporter			**1.6**
LSA0064	*lsa0064*	Putative nucleobase:cation symporter		-0.8	
LSA0259	*lsa0259*	Pyrimidine-specific nucleoside symporter	**1.5**		**1.3**
LSA0798*	*lsa0798*	Pyrimidine-specific nucleoside symporter	**3.5**	**2.2**	**1.7**
LSA0799*	*lsa0799*	Putative purine transport protein	**4.4**	**2.7**	**2.9**
LSA1210	*lsa1210*	Putative cytosine:cation symporter (C-terminal fragment), authentic frameshift	-0.8		-0.6
LSA1211	*lsa1211*	Putative cytosine:cation symporter (N-terminal fragment), authentic frameshit	**-1.1**		-0.9
**Metabolism of nucleotides and nucleic acids**					
LSA0010	*lsa0010*	Putative nucleotide-binding phosphoesterase			-0.6
LSA0023	*lsa0023*	Putative ribonucleotide reductase (NrdI-like)	-0.5	D	D
LSA0063	*purA*	Adenylosuccinate synthetase (IMP-aspartate ligase)		-0.8	
LSA0139	*guaA*	Guanosine monophosphate synthase (glutamine amidotransferase)		-0.5	-0.8
LSA0252	*iunH1*	Inosine-uridine preferring nucleoside hydrolase	**2.6**	**2.6**	**1.8**
LSA0446	*pyrDB*	Putative dihydroorotate oxidase, catalytic subunit			0.9
LSA0489	*lsa0489*	Putative metal-dependent phosphohydrolase precursor	0.5		
LSA0533*	*iunH2*	Inosine-uridine preferring nucleoside hydrolase	**1.2**		
LSA0785	*lsa0785*	Putative NCAIR mutase, PurE-related protein	**-2.3**		**-1.3**
LSA0795*	*deoC*	2 Deoxyribose-5 phosphate aldolase	**4.0**	**2.1**	**2.2**
LSA0796*	*deoB*	Phosphopentomutase (phosphodeoxyribomutase)	**5.5**	**4.1**	**3.2**
LSA0797*	*deoD*	Purine-nucleoside phosphorylase	**4.5**	**2.6**	**1.9**
LSA0801*	*pdp*	Pyrimidine-nucleoside phosphorylase	**1.8**		
LSA0940	*nrdF*	Ribonucleoside-diphosphate reductase, beta chain		**1.0**	0.6
LSA0941	*nrdE*	Ribonucleoside-diphosphate reductase, alpha chain		**1.0**	0.6
LSA0942	*nrdH*	Ribonucleotide reductase, NrdH-redoxin		**1.1**	
LSA0950	*pyrR*	Bifunctional protein: uracil phosphoribosyltransferase and pyrimidine operon transcriptional regulator	-0.6		
LSA0993	*rnhB*	Ribonuclease HII (RNase HII)			0.6
LSA1018	*cmk*	Cytidylate kinase			0.6
LSA1097	*lsa1097*	Putative ADP-ribose phosphorylase, NUDIX family	0.5		
LSA1352	*lsa1352*	Putative phosphomethylpyrimidine kinase	-0.8		
LSA1651	*lsa1651*	Putative purine phosphoribosyltransferase, PRT family		0.8	
LSA1661	*lsa1661*	Putative nucleotide hydrolase, NUDIX family		-0.5	
LSA1805	*dgk*	Deoxyguanosine kinase	**-1.0**		-0.8
					
**Transcription**					
**Transcription regulation**					
LSA0130	*lsa0130*	Putative transcriptional regulator, LacI family	-0.6		
LSA0132	*lsa0132*	Putative transcriptional regulator, MarR family	-0.6		
LSA0161	*lsa0161*	Putative transcriptional regulator, ArsR family	-0.6		
LSA0186	*lsa0186*	Putative transcriptional regulator, LytR family		0.8	0.6
LSA0203	*rbsR*	Ribose operon transcriptional regulator, LacI family	**1.7**		
LSA0217	*lsa0217*	Putative thiosulfate sulfurtransferase with a ArsR-HTH domain, rhodanese family		**-1.0**	-0.7
LSA0229	*lsa0229*	Putative transcriptional regulator, MerR family (N-terminal fragment), authentic frameshift	-0.5		
LSA0269	*lsa0269*	Putative transcriptional regulator, TetR family			-0.6
LSA0293	*lsa0293*	Putative DNA-binding protein, XRE family			-0.6
LSA0356	*rex1*	Redox-sensing transcriptional repressor, Rex	-0.8	-0.5	-0.9
LSA0603	*cggR*	Glycolytic genes regulator		-0.6	-0.6
LSA0669	*lsa0669*	Putative transcription regulator, TetR family		-0.6	
LSA0783	*lsa0783*	Putative transcriptional regulator, Fnr/Crp Family	-0.6		
LSA0800	*deoR*	Deoxyribonucleoside synthesis operon transcriptional regulator, GntR family	**3.8**	**2.1**	**1.9**
LSA0835	*lsa0835*	Putative DNA-binding protein, XRE family	-0.6		
LSA0848	*rex*	Redox-sensing transcriptional repressor, Rex	**1.6**	0.7	
LSA0972	*lsa0972*	Putative transcriptional regulator, LysR family	0.9		
LSA1201	*lsa1201*	Putative transcriptional regulator, GntR family	**1.4**	D	D
LSA1322	*glnR*	Glutamine synthetase transcriptional regulator, MerR family	**-1.4**	**-1.3**	
LSA1351	*lsa1351*	Putative transcritional regulator with aminotransferase domain, GntR family		-0.5	-0.6
LSA1434	*lsa1434*	Putative transcriptional regulator, DUF24 family (related to MarR/PadR families)	-0.8		
LSA1449	*spxA*	Transcriptional regulator Spx	**1.0**		0.6
LSA1521	*lsa1521*	Putative transcriptional regulator, TetR family	0.6		
LSA1554	*lsa1554*	Putative transcriptional regulator, LacI family	-0.7	-0.9	-0.5
LSA1587	*lsa1587*	Putative transcriptional regulator, GntR family	0.6		
LSA1611	*lsa1611*	Putative DNA-binding protein, PemK family		-0.5	-0.7
LSA1653	*lsa1653*	Putative transcriptional regulator, MarR family			-0.6
LSA1692	*lsa1692*	Putative transcriptional regulator, GntR family	0.7		0.7
					
**CoEnzyme transport and metabolism**					
**Metabolism of coenzymes and prostethic groups**					
LSA0041	*panE*	2-dehydropantoate 2-reductase		0.8	
LSA0057	*thiE*	Thiamine-phosphate pyrophosphorylase (thiamine-phosphate synthase)			**1.9**
LSA0058	*thiD*	Phosphomethylpyrimidine kinase (HMP-phosphate kinase)			**1.4**
LSA0059	*thiM*	Hydroxyethylthiazole kinase (4-methyl-5-beta-hydroxyethylthiazole kinase)	**1.0**		**1.8**
LSA0183	*lsa0183*	Putative hydrolase, isochorismatase/nicotamidase family	-0.7		
LSA0840	*lsa0840*	Putative glutamate-cysteine ligase	0.6		
LSA0947	*fhs*	Formate-tetrahydrofolate ligase (formyltetrahydrofolate synthetase)	0.6		
LSA0980	*lsa0980*	Putative hydroxymethylpyrimidine/phosphomethylpyrimidine kinase, PfkB family	0.6		
LSA1101	*folK*	2-amino-4-hydroxy-6-hydroxymethyldihydropteridine pyrophosphokinase	0.6	U	
LSA1614	*acpS*	Holo-[acyl-carrier protein] synthase (holo-ACP synthase) (4'-phosphopantetheine transferase AcpS)	**-1.0**	-0.9	-0.9
LSA1664	*lsa1664*	Putative dihydrofolate reductase	**1.6**	**1.1**	**1.5**
					
**Energy production and conversion**					
**Membrane bioenergetics (ATP synthase)**					
LSA1125	*atpC*	H(+)-transporting two-sector ATPase (ATP synthase), epsilon subunit	0.6		
LSA1126	*atpD*	H(+)-transporting two-sector ATPase (ATP synthase), beta subunit			0.6
LSA1127	*atpG*	H(+)-transporting two-sector ATPase (ATP synthase), gamma subunit			0.8
LSA1128	*atpA*	H(+)-transporting two-sector ATPase (ATP synthase), alpha subunit			0.6
LSA1129	*atpH*	H(+)-transporting two-sector ATPase (ATP synthase), delta subunit			0.6
LSA1130	*atpF*	H(+)-transporting two-sector ATPase (ATP synthase), B subunit			0.5
LSA1131	*atpE*	H(+)-transporting two-sector ATPase (ATP synthase), C subunit			0.7
					
**Inorganic ion transport and metabolism**					
**Transport/binding of inorganic ions**					
LSA0029	*lsa0029*	Putative ion Mg(2+)/Co(2+) transport protein, hemolysinC-family			-0.7
LSA0134	*lsa0134*	Putative Na(+)/H(+) antiporter			-0.6
LSA0180	*mtsC*	Manganese ABC transporter, ATP-binding subunit	-0.8		
LSA0181	*mtsB*	Manganese ABC transporter, membrane-spanning subunit	-0.8		**-1.0**
LSA0182	*mtsA*	Manganese ABC transporter, substrate-binding lipoprotein precursor	-0.7		-0.6
LSA0246	*mntH1*	Mn(2+)/Fe(2+) transport protein	-0.9		**-1.3**
LSA0283	*lsa0283*	Putative zinc/iron ABC transporter, ATP-binding subunit			-0.5
LSA0284	*lsa0284*	Putative zinc/iron ABC transporter, membrane-spanning subunit			-0.6
LSA0399	*lsa0399*	Iron(III)-compound ABC transporter, substrate-binding lipoprotein precursor	**1.1**	0.9	
LSA0400	*lsa0400*	Iron(III)-compound ABC transporter, ATP-binding subunit		0.7	
LSA0401	*lsa0401*	Iron(III)-compound ABC transporter, membrane-spanning subunit			0.5
LSA0402	*lsa0402*	Iron(III)-compound ABC transporter, membrane-spanning subunit	0.5		0.6
LSA0503	*pstC*	Phosphate ABC transporter, membrane-spanning subunit	0.5		
LSA0504	*pstA*	Phosphate ABC transporter, membrane-spanning subunit	0.6		
LSA0781	*lsa0781*	Putative cobalt ABC transporter, membrane-spanning/permease subunit	-0.9		
LSA0782	*lsa0782*	Putative cobalt ABC transporter, membrane-spanning/permease subunit	**-2.1**		
LSA1166	*lsa1166*	Putative potassium transport protein	0.7		
LSA1440	*cutC*	Copper homeostasis protein, CutC family	-0.6		
LSA1460	*atkB*	Copper-transporting P-type ATPase	0.6		
LSA1638	*lsa1638*	Putative large conductance mechanosensitive channel		**-1.0**	-0.8
LSA1645	*lsa1645*	Putative Na(+)/(+) antiporter	**1.4**		D
LSA1699	*mntH2*	Mn(2+)/Fe(2+) transport protein			-0.6
LSA1703	*lsa1703*	Putative Na(+)/H(+) antiporter	**-1.2**		
LSA1704	*lsa1704*	Putative calcium-transporting P-type ATPase			-0.8
LSA1735	*lsa1735*	Putative cobalt ABC transporter, membrane-spanning subunit			-0.6
LSA1736	*lsa1736*	Putative cobalt ABC transporter, ATP-binding subunit	-0.6		
LSA1737	*lsa1737*	Putative cobalt ABC transporter, ATP-binding subunit	-0.7		
LSA1838	*lsa1838*	Putative metal ion ABC transporter, membrane-spanning subunit			-0.5
LSA1839	*lsa1839*	Putative metal ion ABC transporter, substrate-binding lipoprotein precursor			-0.6
					
**Amino acid transport and metabolism**					
**Transport/binding of amino acids**					
LSA0125	*lsa0125*	Putative amino acid/polyamine transport protein	0.6		
LSA0189	*lsa0189*	Putative amino acid/polyamine transport protein			-0.7
LSA0311	*lsa0311*	Putative glutamate/aspartate:cation symporter	**-1.1**		**-1.0**
LSA1037	*lsa1037*	Putative amino acid/polyamine transport protein	**1.0**	0.8	0.5
LSA1219	*lsa1219*	Putative cationic amino acid transport protein	0.7		
LSA1415	*lsa1415*	Putative amino acid/polyamine transport protein	**1.1**		0.7
LSA1424	*lsa1424*	Putative L-aspartate transport protein	**-1.4**	-0.9	**-1.2**
LSA1435	*lsa1435*	Putative amino acid:H(+) symporter	**1.0**		0.8
LSA1496	*lsa1496*	Putative glutamine/glutamate ABC transporter, ATP-binding subunit		**1.2**	
LSA1497	*lsa1497*	Putative glutamine/glutamate ABC transporter, membrane-spanning/substrate-binding subunit precursor		0.7	
**Transport/binding of proteins/peptides**					
LSA0702	*oppA*	Oligopeptide ABC transporter, substrate-binding lipoprotein precursor		**1.3**	**1.0**
LSA0703	*oppB*	Oligopeptide ABC transporter, membrane-spanning subunit		0.8	0.8
LSA0704	*oppC*	Oligopeptide ABC transporter, membrane-spanning subunit		**1.8**	**1.0**
LSA0705	*oppD*	Oligopeptide ABC transporter, ATP-binding subunit		**1.2**	**1.1**
LSA0706	*oppF*	Oligopeptide ABC transporter, ATP-binding subunit		**1.2**	**1.2**
**Protein fate**					
LSA0053	*pepO*	Endopeptidase O	0.6		
LSA0133	*pepR*	Prolyl aminopeptidase	**1.5**		
LSA0226	*pepN*	Aminopeptidase N (lysyl-aminopeptidase-alanyl aminopeptidase)			-0.7
LSA0285	*pepF1*	Oligoendopeptidase F1			-0.7
LSA0320	*pepD3*	Dipeptidase D-type (U34 family)		-0.8	-0.5
LSA0424	*pepV*	Xaa-His dipeptidase V (carnosinase)	**1.6**		
LSA0643	*pepX*	X-Prolyl dipeptidyl-aminopeptidase	0.6		
LSA0888	*pepT*	Tripeptide aminopeptidase T	0.6		
LSA1522	*pepS*	Aminopeptidase S	0.5		
LSA1686	*pepC1N*	Cysteine aminopeptidase C1 (bleomycin hydrolase) (N-terminal fragment), authentic frameshift		**1.6**	
LSA1688	*pepC2*	Cysteine aminopeptidase C2 (bleomycin hydrolase)		0.7	
LSA1689	*lsa1689*	Putative peptidase M20 family	**1.0**		**1.1**
**Metabolism of amino acids and related molecules**					
LSA0220_c	*dapE*	Succinyl-diaminopimelate desuccinylase	**-1.4**		**-1.5**
LSA0316	*sdhB*	L-serine dehydratase, beta subunit (L-serine deaminase)	-0.7		
LSA0370*	*arcA*	Arginine deiminase (arginine dihydrolase)	**1.9**		
LSA0372*	*arcC*	Carbamate kinase	0.5		
LSA0463	*lsa0463*	Putative 2-hydroxyacid dehydrogenase	-0.7		
LSA0509	*kbl*	2-amino-3-ketobutyrate coenzyme A ligase (glycine acetyltransferase)	**1.5**		
LSA0510	*lsa0510*	L-threonine dehydrogenase (N-terminal fragment), authentic frameshift	**2.0**	0.5	
LSA0572*	*tdcB*	Threonine deaminase (threonine ammonia-lyase, threonine dehydratase, IlvA homolog)	**2.2**		**1.7**
LSA0922	*serA*	D-3-phosphoglycerate dehydrogenase	0.9		
LSA1134	*glyA*	Glycine/Serine hydroxymethyltransferase		0.7	
LSA1321	*glnA*	Glutamate-ammonia ligase (glutamine synthetase)	**-1.3**	**-1.0**	
LSA1484	*mvaS*	Hydroxymethylglutaryl-CoA synthase	-0.7	-0.6	-0.7
LSA1693	*asnA2*	L-asparaginase	0.8		
					
**Lipid transport and metabolism**					
**Metabolism of lipids**					
LSA0045	*cfa*	Cyclopropane-fatty-acyl-phospholipid synthase	**-1.3**	**-1.4**	**-1.4**
LSA0644	*lsa0644*	Putative acyl-CoA thioester hydrolase	0.6		
LSA0812	*fabZ1*	(3R)-hydroxymyristoyl-[acyl-carrier protein] dehydratase		-0.7	0.5
LSA0813	*fabH*	3-oxoacyl-[acyl carrier protein] synthetase III			0.6
LSA0814	*acpP*	Acyl carrier protein			0.6
LSA0815	*fabD*	Malonyl-CoA:ACP transacylase		-0.7	0.7
LSA0816	*fabG*	3-oxoacyl-acyl carrier protein reductase		-0.7	
LSA0817	*fabF*	3-oxoacyl-[acyl carrier protein] synthetase II		-0.7	
LSA0819	*fabZ*	(3R)-hydroxymyristoyl-[acyl carrier proetin] dehydratase			0.7
LSA0820	*accC*	Acetyl-CoA carboxylase (biotin carbooxylase subunit)		-0.7	
LSA0821	*accD*	Acetyl-CoA carboxylase (carboxyl transferase beta subunit)			0.8
LSA0822	*accA*	Acetyl-CoA carboxylase (carboxyl transferase alpha subunit)			0.6
LSA0823	*fabI*	Enoyl [acyl carrier protein] reductase			0.9
LSA0891	*lsa0891*	Putative lipase/esterase	1.2		
LSA1485	*mvaA*	Hydroxymethylglutaryl-CoA reductase	-0.5		
LSA1493	*lsa1493*	Putative diacylglycerol kinase	-0.6	-0.9	-0.7
LSA1652	*ipk*	4-diphosphocytidyl-2-C-methyl-D-erythritol kinase	-0.6		-0.7
					
**Secondary metabolites transport and metabolism**					
**Transport/binding proteins and lipoproteins**					
LSA0046	*lsa0046*	Putative transport protein	**-1.0**	-0.6	**-1.3**
LSA0089	*lsa0089*	Putative drug transport protein	**-2.1**	-0.9	-0.8
LSA0094	*lsa0094*	Putative transport protein, Major Facilitator Super (MFS) family transporter	-0.7		-0.7
LSA0095	*lsa0095*	Putative transport protein	**1.3**	0.5	
LSA0128	*lsa0128*	Putative antimicrobial peptide ABC exporter, membrane-spanning/permease subunit			-0.5
LSA0187	*lsa0187*	Putative drug-resistance ABC transporter, two ATP-binding subunits		0.7	
LSA0219_b	*lsa0219_b*	Putative cyanate transport protein	-0.6		
LSA0232	*lmrA*	Multidrug ABC exporter, ATP-binding and membrane-spanning/permease subunits	-0.7		-0.7
LSA0270	*lsa0270*	Putative multidrug ABC exporter, membrane-spanning/permease subunit	-0.7		
LSA0271	*lsa0271*	Putative multidrug ABC exporter, ATP-binding subunit	-0.7		-0.6
LSA0272	*lsa0272*	Putative multidrug ABC exporter, ATP-binding and membrane-spanning/permease subunits	-0.6		-0.6
LSA0308	*lsa0308*	Putative drug:H(+) antiporter			-0.7
LSA0376	*lsa0376*	Putative transport protein	0.7		
LSA0420	*lsa0420*	Putative drug:H(+) antiporter (N-terminal fragment), authentic frameshift	-0.8		**-1.1**
LSA0469	*lsa0469*	Putative drug:H(+) antiporter	-0.6		-0.5
LSA0788	*lsa0788*	Putative facilitator protein, MIP family	**-2.6**		
LSA0936	*lsa0936*	Putative drug ABC exporter, membrane-spanning/permease subunit	**1.1**		
LSA0937	*lsa0937*	Putative drug ABC exporter, membrane-spanning/permease subunit	**1.3**		
LSA0938	*lsa0938*	Putative drug ABC exporter, ATP-binding subunit	**1.2**		
LSA0963	*lsa0963*	Integral membrane protein, hemolysin III related			
LSA1088	*lsa1088*	Putative multidrug ABC exporter, ATP-binding and membrane-spanning/permease subunits	0.5		
LSA1261	*lsa1261*	Putative autotransport protein	0.5		
LSA1340	*lsa1340*	Putative transport protein		-0.7	
LSA1366	*lsa1366*	Putative ABC exporter, ATP-binding subunit	-0.8		**-1.0**
LSA1367	*lsa1367*	Putative ABC exporter, membrane-spanning/permease subunit	-0.8	-0.5	-0.8
LSA1420	*lsa1417*	Putative lipase/esterase		**-1.1**	
LSA1621	*lsa1621*	Putative drug:H(+) antiporter		**-1.1**	
LSA1642	*lsa1642*	Putative Solute:Na(+) symporter	**3.4**	**1.8**	D
LSA1872	*lsa1872*	Putative drug:H(+) antiporter		0.7	
LSA1878	*lsa1878*	Putative drug resistance ABC transporter, two ATP-binding subunits	-0.6		
**Detoxification**					
LSA0772	*lsa0772*	Hypothetical protein (TelA, telluric resistance family)	**1.0**		0.7
LSA1317	*lsa1317*	Putative chromate reductase	0.6	-0.7	
LSA1450	*lsa1450*	Putative metal-dependent hydrolase (beta-lactamase family III)			0.6
LSA1776	*lsa1776*	Putative 4-carboxymuconolactone decarboxylase	0.6		D
					
**Translation, ribosomal structure and biogenesis**					
**Translation initiation**					
LSA1135	*lsa1135*	Putative translation factor, Sua5 family		0.7	0.6
**Translation elongation**					
LSA0251	*efp1*	Elongation factor P (EF-P)	0.5		
LSA1063	*tuf*	Elongation factor Tu (EF-Tu)	0.6		
**Ribosomal proteins**					
LSA0011	*rplI*	50S Ribosomal protein L9			-0.8
LSA0266	*rpsN*	30S ribosomal protein S14		0.7	-0.5
LSA0494	*lsa0494*	30S ribosomal interface protein S30EA	**1.7**		
LSA0696	*rpmB*	50S ribosomal protein L28			0.8
LSA1017	*rpsA*	30S Ribosomal protein S1	0.9		0.6
LSA1333	*rpmG*	50S ribosomal protein L33			0.6
LSA1666	*rplL*	50S ribosomal protein L7/L12	-0.6		
LSA1676	*rpmG2*	50S ribosomal protein L33			-0.6
LSA1750	*rplF*	50S ribosomal protein L6		0.6	
LSA1755	*rpsQ*	30S ribosomal protein S17		0.5	
LSA1761	*rplB*	50S ribosomal protein L2		0.6	
LSA1765	*rpsJ*	30S ribosomal protein S10	-0.7		
**Protein synthesis**					
LSA0377	*tgt*	Queuine tRNA-ribosyltransferase	-0.6		
LSA1546	*gatB*	Glutamyl-tRNA amidotransferase, subunit B		-0.5	
LSA1547	*gatA*	Glutamyl-tRNA amidotransferase, subunit A	-0.5		-0.5
**RNA restriction and modification**					
LSA0437	*lsa0437*	Hypothetical protein with an RNA-binding domain	-0.7		
LSA0443	*lsa0443*	Putative single-stranded mRNA endoribonuclease	**2.7**		**1.9**
LSA0738	*dtd*	D-tyrosyl-tRNA(tyr) deacylase	0.5		
LSA0794	*trmU*	tRNA (5-methylaminomethyl-2-thiouridylate)-methyltransferase		-0.9	
LSA1534	*lsa1534*	Putative ATP-dependent RNA helicase		0.9	
LSA1615	*lsa1615*	Putative ATP-dependent RNA helicase	-0.7	-0.8	**-1.0**
LSA1723	*truA*	tRNA pseudouridylate synthase A (pseudouridylate synthase I)	-0.7		-0.6
LSA1880	*trmE*	tRNA modification GTPase trmE	-0.7		
**Aminoacyl-tRNA synthetases**					
LSA0880	*glyQ*	Glycyl-tRNA synthetase, alpha subunit		0.7	
LSA0881	*glyS*	Glycyl-tRNA synthetase, beta subunit		0.7	
LSA1400	*thrS*	Threonyl-tRNA synthetase	0.6		
LSA1681	*cysS*	Cysteinyl-tRNA synthetase	-0.6		
					
**DNA replication, recombination and repair**					
**DNA replication**					
LSA0221	*lsa0221*	Putative transcriptional regulator, LysR family (C-terminal fragment), degenerate	-0.8	-0.9	**-1.1**
LSA0976	*parE*	Topoisomerase IV, subunit B		0.5	
**Transposon and IS**					
LSA1152_a	*tnpA3-ISLsa1*	Transposase of ISLsa1 (IS30 family)	-0.6		
**Phage-related function**					
LSA1292	*lsa1292*	Putative prophage protein	0.6		
LSA1788	*lsa1788*	Putative phage-related 1,4-beta-N-acetyl muramidase (cell wall hydrolase)	**-1.0**	D	D
**DNA recombination and repair**					
LSA0076	*lsa0076*	Putative DNA invertase (plasmidic resolvase)	**-1.1**	**-1.5**	**-1.4**
LSA0366	*ruvA*	Holliday junction DNA helicase RuvA			-0.5
LSA0382	*dinP*	DNA-damage-inducible protein P	-0.5		
LSA0487	*recA*	DNA recombinase A	-0.8		**-1.1**
LSA0523	*uvrB*	Excinuclease ABC, subunit B	-0.7		-0.5
LSA0524	*uvrA1*	Excinuclease ABC, subunit A	**-1.2**		-0.7
LSA0910	*rexAN*	ATP-dependent exonuclease, subunit A (N-terminal fragment), authentic frameshift	0.6		
LSA0911	*rexAC*	ATP-dependent exonuclease, subunit A (C-terminal fragment), authentic frameshift	0.7		
LSA0912	*lsa0912*	Putative ATP-dependent helicase, DinG family	0.6		0.8
LSA1162	*lsa1162*	DNA-repair protein (SOS response UmuC-like protein)		0.8	-0.6
LSA1405	*fpg*	Formamidopyrimidine-DNA glycosylase	-0.5	-0.6	-0.6
LSA1477	*recX*	Putative regulatory protein, RecX family	-0.6		
LSA1843	*ogt*	Methylated-DNA-protein-cysteine S-methyltransferase	-0.6		
**DNA restriction and modification**					
LSA0143	*lsa0143*	Putative adenine-specific DNA methyltransferase	-0.7	D	D
LSA0921	*lsa0921*	Putative adenine-specific DNA methyltransferase	0.8		
LSA1299	*lsa1299*	Putative adenine-specific DNA methyltransferase	0.9	0.7	**1.2**
**Information pathways**					
LSA0326	*lsa0326*	Putative DNA helicase		-0.6	U
**DNA packaging and segregation**					
LSA0135	*lsa0135*	Hypothetical integral membrane protein, similar to CcrB			-0.6
LSA1015	*hbsU*	Histone-like DNA-binding protein HU	**1.0**		0.9
					
**Cell division and chromosome partitioning**					
**Cell division**					
LSA0755	*divIVA*	Cell-division initiation protein (septum placement)			0.5
LSA0845	*lsa0845*	Putative negative regulator of septum ring formation	0.7		0.6
LSA1118	*lsa1118*	Rod-shape determining protein		0.6	0.5
LSA1597	*ftsH*	ATP-dependent zinc metalloendopeptidase FtsH (cell division protein FtsH)			-0.6
LSA1879	*gidA*	Cell division protein GidA	-0.6		
					
**Cell envelope biogenesis, outer membrane**					
**Cell wall**					
LSA0280	*murE*	UDP-N-acetylmuramoylalanyl-D-glutamate-2,6-diaminopimelate ligase	-0.6	-0.6	-0.7
LSA0621	*pbp2A*	Bifunctional glycolsyltransferase/transpeptidase penicillin binding protein 2A			0.7
LSA0648	*lsa0648*	Putative penicillin-binding protein precursor (beta-lactamase class C)			**1.0**
LSA0862	*lsa0862*	N-acetylmuramoyl-L-alanine amidase precursor (cell wall hydrolase) (autolysin)	0.6		0.8
LSA0917	*pbp1A*	Bifunctional glycosyltransferase/transpeptidase penicillin-binding protein 1A			0.5
LSA1123	*murA1*	UDP-N-acetylglucosamine 1-carboxyvinyltransferase I		-0.5	
LSA1334	*pbp2B2*	Bifuntional dimerisation/transpeptidase penicillin-binding protein 2B		0.7	0.7
LSA1437	*lsa1437*	N-acetylmuramoyl-L-alanine amidase precursor (cell wall hydrolase) (autolysin)		-0.7	
LSA1441	*bacA*	Putative undecaprenol kinase (bacitracine resistance protein A)		0.6	
LSA1613	*alr*	Alanine racemase	-0.8	-0.9	-0.7
LSA1616	*murF*	UDP-N-acetylmuramoyl-tripeptide--D-alanyl-D-alanine ligase			-0.5
**Cell envelope and cellular processes**					
LSA0162	*lsa0162*	Putative Bifunctional glycosyl transferase, family 8		**-1.2**	**-1.5**
LSA1246	*lsa1246*	Putative glycosyl transferase, family 2		-0.9	
LSA1558	*lsa1558*	Putative extracellular N-acetylmuramoyl-L-alanine amidase precursor (cell wall hydrolase/Lysosyme subfamily 2)			-0.6
					
**Cell motility and secretion**					
**Protein secretion**					
LSA0948	*lspA*	Signal peptidase II (lipoprotein signal peptidase) (prolipoprotein signal peptidase)			0.5
LSA1884	*oxaA2*	Membrane protein chaperone oxaA			-0.6
					
**Signal transduction**					
**Signal transduction**					
LSA0561	*sppKN*	Two-component system, sensor histidine kinase, (SppK fragment), degenerate		0.5	
LSA0692	*lsa0692*	Putative serine/threonine protein kinase		0.5	0.6
LSA1384	*lsa1384*	Two-component system, response regulator		0.5	
					
**Post translational modifications, protein turnover, chaperones**					
**Protein folding**					
LSA0050	*lsa0050*	Putative molecular chaperone, small heat shock protein, Hsp20 family			-0.7
LSA0082	*htrA*	Serine protease HtrA precursor, trypsin family		-0.6	
LSA0207	*clpL*	ATPase/chaperone ClpL, putative specificity factor for ClpP protease	0.6		
LSA0358	*groS*	Co-chaperonin GroES (10 kD chaperonin) (protein Cpn10)			-0.5
LSA0359	*groEL*	Chaperonin GroEL (60 kDa chaperonin) (protein Cpn60)			-0.5
LSA0436	*lsa0436*	Putative peptidylprolyl isomerase (peptidylprolyl cis-trans isomerase) (PPIase)			-0.6
LSA0984	*hslU*	ATP-dependent Hsl protease, ATP-binding subunit HslU	0.7		0.7
LSA1465	*clpE*	ATPase/chaperone ClpE, putative specificity factor for ClpP protease	-0.7	-0.6	-0.6
LSA1618	*htpX*	Membrane metalloprotease, HtpX homolog		0.8	
**Adaption to atypical conditions**					
LSA0170	*lsa0170*	Putative general stress protein	0.5		**-1.5**
LSA0247	*usp2*	Similar to universal stress protein, UspA family			-0.5
LSA0264	*lsa0264*	Putative glycine/betaine/carnitine/choline transport protein	-0.6		-0.6
LSA0513	*lsa0513*	Putative stress-responsive transcriptional regulator		-0.8	
LSA0552	*lsa0552*	Organic hydroperoxide resistance protein		0.6	
LSA0616	*lsa0616*	Putative glycine/betaine/carnitine/choline ABC transporter, ATP-binding subunit	0.9		
LSA0617	*lsa0617*	Putative glycine/betaine/carnitine/choline ABC transporter, membrane-spanning subunit	**1.3**		
LSA0618	*lsa0618*	Putative glycine/betaine/carnitine/choline ABC transporter, substrate-binding lipoprotein	0.6		
LSA0619	*lsa0619*	Putative glycine/betaine/carnitine/choline ABC transporter, membrane-spanning subunit	**1.5**	0.5	
LSA0642	*usp3*	Similar to universal stress protein, UspA	0.9		
LSA0768	*csp1*	Similar to cold shock protein, CspA family	**2.1**	0.6	**1.8**
LSA0836	*usp6*	Similar to universal stress protein, UspA family	0.6		
LSA0946	*csp4*	Similar to cold shock protein, CspA family	0.6		
LSA1110	*lsa1110*	Putative NifU-homolog involved in Fe-S cluster assembly		0.6	
LSA1111	*lsa1111*	Putative cysteine desulfurase (class-V aminotransferase, putative SufS protein homologue)		0.7	
LSA1173	*usp4*	Similar to universal stress protein, UspA family	**1.5**	**-2.1**	
LSA1694	*lsa1694*	Putative glycine/betaine/carnitine ABC transporter, substrate binding lipoprotein precursor	**-1.7**		**-1.1**
LSA1695	*lsa1695*	Putative glycine/betaine/carnitine ABC transporter, membrane-spanning subunit	**-2.1**	**-2.0**	**-1.9**
LSA1696	*lsa1696*	Putative glycine/betaine/carnitine ABC transporter, ATP-binding subunit	**-1.6**		-0.9
LSA1870	*lsa1870*	Putative glycine betaine/carnitine/choline ABC transporter, ATP-binding subunit	-0.6		-0.6
**Protein modification**					
LSA0865	*lsa0865*	Putative protein methionine sulfoxide reductase		-0.6	
LSA0866	*msrA*	Protein methionine sulfoxide reductase		-0.7	
LSA0934	*lplA*	Lipoate-protein ligase	**1.6**	**1.4**	**1.0**
LSA0973	*pflA*	Pyruvate formate-lyase activating enzyme	**1.7**		
					
**General function prediction only**					
**Miscellaneous**					
LSA0030	*lsa0030*	Putative aldo/keto reductase (oxidoreductase)		-0.7	-0.8
LSA0120	*lsa0120*	Putative GTP-binding protein	-0.5		
LSA0164	*lsa0164*	Putative serine/tyrosine protein phosphatase	0.2	**-1.1**	**-1.2**
LSA0165	*lsa0165*	Putative oxidoreductase, short chain dehydrogenase/reductase family		-0.9	**-1.2**
LSA0218	*trxA1*	Thioredoxin		-0.9	
LSA0258	*lsa0258*	Putative iron-containing alcohol dehydrogenase	**1.6**	0.5	**1.6**
LSA0260	*lsa0260*	Putative aldo/keto reductase (oxidoreductase)	**1.9**	1.2	**1.7**
LSA0312	*lsa0312*	Putative NADH oxidase	-0.9		**-1.0**
LSA0324	*lsa0324*	Putative hydrolase, haloacid dehalogenase family (N-terminal fragment), authentic frameshift	**1.9**		
LSA0325	*lsa0325*	Putative hydrolase, haloacid dehalogenase family (C-terminal fragment), authentic frameshift	**1.8**		
LSA0350	*lsa0350*	Putative N-acetyltransferase, GNAT family	-0.5		
LSA0369	*lsa0369*	Putative N-acetyltransferase, GNAT family	-0.5		-0.5
LSA0384	*lsa0384*	Putative phosphoesterase, DHH family	-0.5		
LSA0403	*lsa0403*	Putative thioredoxin reductase		0.9	
LSA0447	*lsa0447*	Putative hydrolase, haloacid dehalogenase family			0.6
LSA0475	*lsa0475*	Putative N-acetyltransferase, GNAT family		-0.6	
LSA0520	*trxB2*	Thioredoxin reductase	-0.8		
LSA0575	*npr*	NADH peroxidase	**1.0**	U	
LSA0802	*nox*	NADH oxidase	**1.5**		
LSA0806	*lsa0806*	Putative N-acetyltransferase, GNAT family	0.6		
LSA0831	*lsa0831*	Putative nitroreductase (oxidoreductase)		**1.6**	
LSA0896	*sodA*	Iron/Manganese superoxide dismutase	**3.4**	**1.7**	**1.7**
LSA0925	*adh*	Putative zinc-containg alcohol dehydrogenase (oxidoreductase)	0.5		
LSA0971	*ppa*	Inorganic pyrophosphatase (pyrophosphate phosphohydrolase)	0.7		
LSA0994	*lsa0994*	Putative GTP-binding protein			0.6
LSA1016	*engA*	Putative GTP-binding protein	0.6		0.7
LSA1045	*obgE*	Putative GTP-binding protein	0.6		
LSA1153	*lsa1153*	Hypothetical protein, CAAX protease family	0.5		
LSA1311	*lsa1311*	Hypothetical protein containing a possible heme/steroid binding domain	0.7	-0.6	
LSA1320	*lsa1320*	Putative NADPH-quinone oxidoreductase		-0.8	
LSA1345	*lsa1345*	Putative hydrolase, haloacid dehalogenase family	0.5		
LSA1349	*lsa1349*	Putative N-acetyltransferase, GNAT family		-0.5	
LSA1365	*lsa1365*	Hypothetical protein		-0.5	-0.7
LSA1368	*lsa1368*	Hypothetical protein	0.9		0.6
LSA1371	*lsa1371*	Hypothetical membrane protein	0.6		
LSA1395	*lsa1395*	Putative zinc-containing alcohol dehydrogenase (oxidoreductase)	0.9		
LSA1427	*lsa1427*	Putative hydrolase, haloacid dehalogenase	**1.3**		0.6
LSA1472	*lsa1472*	Putative N-acetyl transferase, GNAT family	0.6		
LSA1535	*lsa1535*	Putative oxidoreductase	0.5	**1.1**	0.7
LSA1553	*lsa1553*	Putative hydrolase, haloacid dehalogenase family	-0.6		
LSA1559	*lsa1559*	Putative oxidoreductase	0.6	**1.1**	0.7
LSA1702	*lsa1702*	Putative zinc-containing alcohol dehydrogenase (oxidoreductase)	**1.1**		
LSA1712	*lsa1712*	Putative nitroreductase (oxidoreductase)		-0.7	-0.8
LSA1832	*lsa1832*	Putative zinc-containing alcohol dehydrogenase (oxidoreductase)		**1.0**	
LSA1835	*lsa1835*	Putative zinc-containing alcohol dehydrogenase (oxidoreductase)	-0.7		**-1.0**
LSA1867	*lsa1867*	Putative acetyltransferase, isoleucine patch superfamily	-0.5	-0.6	-0.7
LSA1871	*gshR*	Glutathione reductase	-0.6		
					
**Unknown**					
**Proteins of unknown function that are similar to other proteins**					
LSA0018	*lsa0018*	Hypothetical protein		0.5	
LSA0027	*lsa0027*	Hypothetical protein			**-1.1**
LSA0028	*lsa0028*	Hypothetical protein, DegV family	-0.5		
LSA0044	*lsa0044*	Hypothetical protein			-0.7
LSA0061	*lsa0061*	Hypothetical extracellular protein precursor	-0.5		
LSA0106	*lsa0106*	Hypothetical cell surface protein precursor	0.5		
LSA0160	*lsa0160*	Hypothetical protein	-0.7		
LSA0166	*lsa0166*	Hypothetical Integral membrane protein			**-1.2**
LSA0190	*lsa0190*	Hypothetical integral membrane protein	-0.7		-0.6
LSA0191	*lsa0191*	Hypothetical integral membrane protein	-0.6		-0.6
LSA0199	*lsa0199*	Hypothetical protein	**1.1**	**1.0**	**1.1**
LSA0208	*lsa0208*	Hypothetical integral membrane protein	0.7		
LSA0235	*lsa0235*	Hypothetical extracellular protein precursor	**2.1**	**1.6**	**1.7**
LSA0236	*lsa0236*	Hypothetical extracellular peptide precursor	**2.0**	**1.3**	**1.5**
LSA0244	*lsa0244*	Hypothetical integral membrane protein			-0.5
LSA0245	*lsa0245*	Hypothetical lipoprotein precursor	-0.9	**-1.0**	**-1.1**
LSA0249	*lsa0249*	Hypothetical protein	**1.1**	**1.0**	
LSA0263	*lsa0263*	Hypothetical integral membrane protein	-0.6		-0.9
LSA0300	*lsa0300*	Hypothetical protein			0.7
LSA0315	*lsa0315*	Hypothetical protein	-0.7		
LSA0319	*lsa0319*	Hypothetical protein		-0.8	-0.8
LSA0323	*lsa0323*	Hypothetical protein			-0.5
LSA0337	*lsa0337*	Hypothetical protein	-0.7		
LSA0348	*lsa0348*	Hypothetical integral membrane protein	-0.9		-0.7
LSA0352	*lsa0352*	Hypothetical integral membrane protein	-0.6		
LSA0354	*lsa0354*	Hypothetical integral membrane protein			**-1.1**
LSA0388	*lsa0388*	Hypothetical protein		-0.6	
LSA0389	*lsa0389*	Hypothetical protein		-0.7	-0.7
LSA0390	*lsa0390*	Hypothetical protein		-0.5	
LSA0409	*lsa0409*	Hypothetical integral membrane protein			-0.8
LSA0418	*lsa0418*	Hypothetical protein			-0.8
LSA0464	*lsa0464*	Hypothetical protein		-0.6	
LSA0470	*lsa0470*	Hypothetical protein	0.9		0.7
LSA0512	*lsa0512*	Hypothetical protein		-0.6	
LSA0515	*lsa0515*	Hypothetical integral membrane protein		-0.5	
LSA0536	*lsa0536*	Hypothetical protein		0.7	
LSA0716	*lsa0716*	Hypothetical protein			0.6
LSA0752	*lsa0752*	Hypothetical protein	0.5		0.6
LSA0757	*lsa0757*	Hypothetical protein		0.8	
LSA0773	*lsa0773*	Hypothetical protein	0.9		0.6
LSA0784	*lsa0784*	Hypothetical protein	**-2.6**		
LSA0786	*lsa0786*	Hypothetical protein	**-2.0**		
LSA0787	*lsa0787*	Hypothetical protein	**-1.7**		
LSA0790	*lsa0790*	Hypothetical protein, ATP utilizing enzyme PP-loop family	**-2.5**		
LSA0827	*lsa0827*	Hypothetical lipoprotein precursor	0.8		U
LSA0828	*lsa0828*	Hypothetical protein	0.7		
LSA0829	*lsa0829*	Hypothetical integral membrane protein			0.5
LSA0874	*lsa0874*	Hypothetical protein	0.5		
LSA0901	*lsa0901*	Hypothetical protein			0.5
LSA0913	*lsa0913*	Hypothetical extracellular protein precursor	0.5		0.7
LSA0919	*lsa0919*	Hypothetical protein			0.7
LSA0933	*lsa0933*	Hypothetical protein	0.6		0.6
LSA0961	*lsa0961*	Hypothetical protein, DegV family		-0.5	
LSA0968	*lsa0968*	Hypothetical integral membrane protein	0.7		
LSA0977	*lsa0977*	Hypothetical integral membrane protein	0.7		0.8
LSA0987	*lsa0987*	Hypotehtical protein, GidA family (C-terminal fragment)	0.5		
LSA0996	*lsa0996*	Hypothetical protein			0.5
LSA1003	*lsa1003*	Hypothetical protein	**2.0**		**1.2**
LSA1005	*lsa1005*	Hypothetical membrane protein	0.9	0.6	0.7
LSA1008	*lsa1008*	Putative extracellular chitin-binding protein precursor		0.9	**1.2**
LSA1027	*lsa1027*	Hypothetical protein			0.6
LSA1047	*lsa1047*	Hypothetical protein	**3.5**	**1.2**	**1.3**
LSA1064	*lsa1064*	Hypothetical protein	0.5		0.7
LSA1075	*lsa1075*	Hypothetical protein			0.5
LSA1078	*lsa1078*	Hypothetical protein			0.6
LSA1081	*lsa1081*	Hypothetical protein	**1.0**		**1.0**
LSA1091	*lsa1091*	Hypothetical protein			0.6
LSA1096	*lsa1096*	Hypothetical protein	0.6		
LSA1124	*lsa1124*	Hypothetical protein		-0.7	
LSA1154	*lsa1154*	Hypothetical protein	0.6		0.6
LSA1158	*lsa1158*	Hypothetical protein	**1.7**	**1.4**	
LSA1189	*lsa1189*	Hypothetical integral membrane protein	**-1.6**		**-1.1**
LSA1282	*lsa1282*	Hypothetical protein		-0.5	
LSA1296	*lsa1296*	Hypothetical integral membrane protein		**-1.2**	-0.8
LSA1342	*lsa1342*	Hypothetical protein		-0.7	
LSA1346	*lsa1346*	Hypothetical protein	0.8		
LSA1350	*lsa1350*	Hypothetical protein		-0.6	**-1.0**
LSA1353	*lsa1353*	Hypothetical integral membrane protein	-0.9	-0.5	
LSA1446	*lsa1446*	Hypothetical protein	-0.6	-0.6	-0.7
LSA1466	*lsa1466*	Hypothetical protein	0.6		
LSA1467	*lsa1467*	Hypothetical protein		-0.6	**-1.1**
LSA1524	*lsa1524*	Hypothetical protein	0.7		
LSA1540	*lsa1540*	Hypothetical extracellular protein precursor	0.7		
LSA1563	*lsa1563*	Hypothetical integral membrane protein		-0.6	-0.6
LSA1610	*lsa1610*	Hypothetical integral membrane protein	-0.7		-0.9
LSA1617	*lsa1617*	Hypothetical protein			-0.7
LSA1620	*lsa1620*	Hypothetical protein			-0.6
LSA1623	*lsa1623*	Hypothetical integral membrane protein	-0.5		-0.6
LSA1637	*lsa1637*	Hypothetical integral membrane protein, TerC family	**-1.7**	**-1.0**	**-1.6**
LSA1644	*lsa1644*	Hypothetical protein	**1.7**		D
LSA1649	*lsa1649*	Hypothetical extracellular protein precursor			-0.5
LSA1659	*lsa1659*	Hypothetical protein	-0.5		
LSA1662	*lsa1662*	Hypothetical protein	**-1.0**	-0.6	-0.7
LSA1663	*lsa1663*	Hypothetical protein	-0.8		
LSA1678	*lsa1678*	Hypothetical protein	-0.6		
LSA1680	*lsa1680*	Hypothetical protein	-0.6		
LSA1716	*lsa1716*	Hypothetical protein		-0.5	
LSA1822	*lsa1822*	Hypothetical protein			-0.5
LSA1828	*lsa1828*	Hypothetical integral membrane protein	0.6	0.7	
LSA1850	*lsa1850*	Hypothetical protein		-0.6	
LSA1876	*lsa1876*	Hypothetical integral membrane protein			-0.6
LSA1877	*lsa1877*	Hypothetical protein			-0.6
**Proteins of unknown function only similar to other proteins from the same organism**					
LSA1159	*lsa1159*	Hypothetical cell surface protein precursor	**2.0**		0.5
LSA1165	*lsa1165*	Hypothetical cell surface protein precursor	**1.8**		
LSA1700	*lsa1700*	Hypothetical protein	**2.1**	0.8	
LSA1814	*lsa1814*	Hypothetical protein			-0.5
**Proteins of unknown function. without similarity to other proteins**					
LSA0065	*lsa0065*	Hypothetical integral membrane protein	-0.5		
LSA0093	*lsa0093*	Hypothetical integral membrane protein	-0.9		**-1.2**
LSA0121	*lsa0121*	Hypothetical small peptide	-0.7	-0.6	-0.5
LSA0163	*lsa0163*	Hypothetical protein		**-1.1**	**-1.3**
LSA0167	*lsa0167*	Hypothetical protein			**-1.4**
LSA0168	*lsa0168*	Hypothetical protein			**-1.4**
LSA0188	*lsa0188*	Hypothetical small peptide			-0.8
LSA0256_a	*lsa0256_a*	Hypothetical protein	**2.3**	**1.0**	**2.2**
LSA0257	*lsa0257*	Hypothetical protein	**1.4**		
LSA0281	*lsa0281*	Hypothetical lipoprotein precursor		-0.5	-0.6
LSA0301	*lsa0301*	Hypothetical protein			0.6
LSA0334	*lsa0334*	Hypothetical extracellular protein precursor	**1.1**		
LSA0339	*lsa0339*	Hypothetical protein	-0.5		
LSA0378	*lsa0378*	Hypothetical protein	-0.7		
LSA0514	*lsa0514*	Hypothetical small extracellular protein precursor		-0.8	
LSA0534	*lsa0534*	Hypothetical cell surface protein precursor (with LPQTG sorting signal)	**1.0**		D
LSA0576	*lsa0576*	Hypothetical protein	0.5	D	
LSA0641	*lsa0641*	Hypothetical extracellular peptide precursor		-0.5	
LSA0647	*lsa0647*	Hypothetical extracellular protein precursor	0.6		
LSA0667	*lsa0667*	Hypothetical protein	**1.0**		0.9
LSA0753	*lsa0753*	Hypothetical integral membrane protein			0.5
LSA0789	*lsa0789*	Hypothetical protein	**-1.9**		
LSA0837	*lsa0837*	Hypothetical protein	**1.2**	**1.3**	**1.4**
LSA0885	*lsa0885*	Hypothetical protein	**1.8**		
LSA0902	*lsa0902*	Hypothetical protein	0.7	D	
LSA0945	*lsa0945*	Hypothetical protein			0.9
LSA1019	*lsa1019*	Hypothetical cell surface protein precursor			0.8
LSA1035	*lsa1035*	Hypothetical small integral membrane protein			0.6
LSA1086	*lsa1086*	hypothetical protein	0.8		0.5
LSA1104	*lsa1104*	Hypothetical protein	-0.5		
LSA1155	*lsa1155*	Hypothetical integral membrane protein	0.5		
LSA1174	*lsa1174*	Hypothetical protein	**1.0**		
LSA1176	*lsa1176*	Hypothetical protein		**-1.0**	U
LSA1319	*lsa1319*	Hypothetical small protein		-0.8	
LSA1408	*lsa1408*	Hypothetical protein			0.6
LSA1464	*lsa1464*	Hypothetical protein	-0.6		
LSA1478	*lsa1478*	Hypothetical protein	-0.7	-0.6	-0.6
LSA1480	*lsa1480*	Hypothetical membrane protein	0.5	D	
LSA1524	*lsa1524*	Hypothetical protein	0.8		
LSA1539	*lsa1539*	Hypothetical protein	0.9		
LSA1713	*lsa1713*	Hypothtical small peptide			-0.6
LSA1787	*lsa1787*	Hypothetical cell surface protein precursor	-0.5	U	
LSA1820	*lsa1820*	Hypothetical cell surface protein precursor			-0.6
LSA1821	*lsa1821*	Hypothetical cell surface protein precursor		-0.6	
LSA1845	*lsa1845*	Hypothetical small protein		0.8	
LSA1848	*lsa1848*	Hypothetical protein			-0.5
LSA1851	*lsa1851*	Hypothetical extracellular small protein	-0.6		-0.7
LSA1883	*lsa1883*	Hypothetical small protein	**1.2**		**1.5**
					
**Bacteriocin associated genes**					
SKP0001	*sppIP*	Bacteriocin sakacin P inducing peptide	D	0.5	D
SKP0006	*sppT*	Sakacin P ABC transporter	D	0.6	D
SKP0007	*sppE*	Sakacin P accesory transport protein	D	0.6	D

The microarray used has been described previously [32]. Asterix (*) relates the gene to Table 2. D and U refer to genes classified as 'divergent' and 'uncertain', respectively, by CGH analysis [32]. Genes encoding proteins with a change in expression according to McLeod et al. [19], are underlined.

Several of the up-regulated genes are located in operons, an organisation believed to provide the advantage of coordinated regulation. In addition, in order to discriminate genes induced by growth on ribose from those repressed by glucose (submitted to CCR mediated by CcpA), a search of the complete genome sequence of *L. sakei *23K [7] was undertaken, with the aim to identify putative *cre *sites. The search revealed 1962 hits, most of which did not have any biological significance considering their unsuitable location in relation to promoters. Relief of CcpA-mediated CCR likely occur for many of the up-regulated genes in the category of carbohydrate transport and metabolism. Putative *cre *sites were identified in their promoter region, as well as for some genes involved in nucleoside and amino acid transport and metabolism (Table 2). In the other gene categories, the presences of putative *cre *sites were rare. With regard to gene product, the *L. sakei *genome shares high level of conservation with *Lactobacillus plantarum *[7], and high similarity of catabolic operon organization. The role of CcpA in CCR in *L. plantarum *has been established, and was shown to mediate regulation of the *pox *genes encoding pyruvate oxidases [41,42]. During growth on ribose, *L. plantarum *induces a similar set of genes as observed in the present study, and putative *cre *sites were identified in the upstream region of several genes involved [33].

**Table 2:** Putative *cre *sites present in the promoter region of some *L. sakei *genes up-regulated in the present study.

Gene locus	Gene	*cre *site sequence^a^	Position^b^	Co-transcribed genes/operon^c^	Gene locus
LSA0123	*lsa0123 *	TGAAAG**CG**TTACAA	-93		
LSA0185	*galP*	GAACAT**CG**TTATCA	-46		
LSA0200	*rbsU*	GTAAAC**CG**TTTTCA	-113	*rbsUDK*	LSA0200-0202
LSA0254	*lsa0254 *	TGTAAG**CG**TTTTAT	-56	*lsa0254-lsa0255-lsa0256_a*	LSA0254-0256_a
LSA0289	*xpk*	CTATTA**CG**ATGACA	-8		
LSA0292	*budC*	TGTAAC**CG**TTTTAA	-51		
LSA0353	*lsa0353 *	AGAAAG**CG**CTTATA	-102		
LSA0370	*arcA*	TGAAAG**CG**ATTACC	-58	*arcA-arcB^e^-arcC-arcT^e^-arcD^e^*	LSA0370-0374
LSA0449	*manL*	TGTTAG**CG**TTTTTA	-56	*manL-manM-manN*	LSA0449-0451
LSA0533	*iunH2 *	AAAAAG**CG**TTCACA	-35		
LSA0572	*tdcB*	TGAAAA**CG**TTCTAA	-134		
LSA0608	*Glo AN*	TGTAAC**CG**TTTTAA	-100	*gloAN-gloAC*	LSA0608-0609
LSA0649	*glpK*	AGGAAA**CG**TTTTCC	-42	*glpK-glpD-glpF*	LSA0649-0651
LSA0664	*loxL1 *	AGAAAG**CG**AGTACA	-82	*loxL1N-loxLI-loxL1C*	LSA0664-0666
LSA0764	*galK*	TGAAAG**CG**ATTAAT	-30	*galK-galE1-galT-galM*	LSA0764-0767
LSA0795	*deoC*	TGAAAG**CG**TTAACA	-33	*deoC-deoB-deoD-lsa0798-lsa0799-deoR-pdp*	LSA0795-0801
LSA0974	*pflB*	TACGAA**CG**CTTACA	-147	*pflB-pflA*	LSA0974-0973
LSA1048	*fruR^e^*	TGTAAA**CG**ATGACA	-39	*fruR^e^-fruK^e^-fruA*	LSA1048-1050
LSA1141	*ppdK *	GGTTAT**CG**ATAAAA	-29		
LSA1146	*manA *	CGAAAT**CG**CTTTAA	-98		
LSA1188	*pox1*	TGTAAT**CG**ATTTCA	-88		
LSA1204	*lsa1204 *	TGTAAT**CG**TTTTTT	-127		
LSA1343	*eutD*	GTAAAA**CG**CTCTCA	-94		
LSA1399	*loxL2*	TGTAAA**CG**ATTTCA	-42		
LSA1457	*lsa1457*	TGATAA**CG**CTTACA	-85		
LSA1463^d^	*ptsH*	TGAAAG**CG**GTATAG	-161	*ptsHI*	LSA1463-1462
LSA1641	*nanE*	TGTAAG**CG**GTTAAT	-85	*nanE-nanA*	LSA1641-1640
LSA1643	*lsa1643*	TGATAA**CG**CTTACA	-31		
LSA1651	*lsa1651 *	GGTAAG**CG**GTTAAA	-148		
LSA1711	*lacL*	TGAAAC**CG**TTTTAA	-36	*lacL-lacM*	LSA1711-1710
LSA1792	*scrA*	TGTAAA**CG**GTTGTA	-78	*scrA-dexB-scrK*	LSA1792-1790
LSA1830	*pox2*	TTGTAA**CG**CTTACA	-70		

The identification is based on the genome sequence of *L. sakei *strain 23K, and the consensus sequence TGWNAN**CG **NTNWCA (W = A/T, N = A/T/G/C), confirmed in Gram-positive bacteria [39] was used in the search, allowing up to two mismatches (underlined) in the conserved positions except for the two center positions, highlighted in boldface.

^a ^mismatch to consensus sequence is underlined

^b ^position of *cre *in relation to the start codon

^c ^suggested co-transcribed genes or genes organized in an operon

^d ^*cre *in preceding gene encoding hypothetical protein

^e ^gene not regulated in this study

### Ribose catabolism and PKP

**Figure 2:**
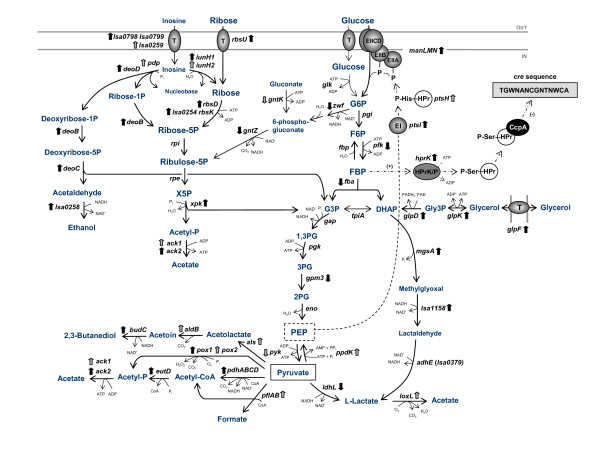
**Overview of the glycolysis, phosphoketolase pathway and nucleoside catabolic pathway affected by the change of carbon source from glucose to ribose in three *L. sakei *strains in this study**. Genes which expression is up- or down-regulated are indicated with upward and downward pointing arrows, respectively, and are listed in Table 1. Black arrows indicate regulation in all three strains, and grey arrows indicate regulation in one or two strains. Schematic representation of CcpA-mediated CCR pathway is shown in the upper right corner. EII, enzyme II of the phosphotransferase system (PTS); EI, enzyme I, HPr, Histidine-containing protein; T, transport protein; P, phosphate; HPrK/P, HPr kinase/phosphatase; G6P, glucose-6-phosphate; F6P; fructose-6-phosphate; FBP, fructose-1,6-bisphosphate; G3P, glyceraldehyde-3-phosphate; DHAP, dihydroxyacetone phosphate; Gly3P, glycerol-3-phosphate; X5P, xylulose-5-phosphate; 1,3PG, 1,3-phosphoglycerate; 3PG, 3-phosphoglycerate; 2PG, 2-phosphoglycerate; PEP, phosphoenolepyruvate; *glk*, glucokinase; *pgi*, phosphoglucoisomerase; *fbp*, fructose-1,6-bisphosphatase; *tpi*, triose-phosphate isomerase; *gap*, glyceraldehyde-3-phosphate dehydrogenase; *pgk*, phosphoglycerate kinase; *eno*, enolase; *rpi*, ribose-5-phosphate isomerase; *rpe*, ribulose-phosphate 3-epimerase.

Confirming its major role in ribose transport and utilization in *L. sakei*, and in agreement with previous findings [16], our microarray data revealed a strong up-regulation (Table 1; log_2 _= 2.8-4.3) of *rbsUDK*. The genes encoding an additional putative carbohydrate kinase belonging to the ribokinase family and a putative phosphoribosyl isomerase, *lsa0254 *and *lsa0255*, respectively, previously suggested to be involved in catabolism of ribose in *L. sakei *[7], were induced in all the strains (Table 1). Recent CGH studies revealed that some *L. sakei *strains which were able to grow on ribose did not harbour the *rbsK *gene, whereas *lsa0254 *was present in all strains investigated [32]. This second ribokinase could therefore function as the main ribokinase in some *L. sakei *strains. The *rbsK *sequence could also differ considerably from that of 23K in these strains. The PKP showed an obvious induction with an up-regulation (2.2-3.2) of the *xpk *gene encoding the key enzyme xylulose-5-phosphate phosphoketolase (Xpk). This enzyme connects the upper part of the PKP to the lower part of glycolysis by converting xylulose-5-phosphate into glyceraldehyde-3-phosphate and acetyl-phosphate. Acetyl-phosphate is then converted to acetate and ATP by acetate kinase (Ack). Supporting our results, previous proteomic analysis showed an over-expression of RbsK, RbsD and Xpk during growth on ribose [15,16,19]. The induction of ribose transport and phosphorylation, and increased phosphoketolase and acetate kinase activities were previously observed during growth on ribose [15]. Three genes encoding Ack are present in the 23K genome [7], as well as in MF1053 and LS 25 [32]. A preferential expression of different *ack *genes for the acetate kinase activity seem to exist. The *ack2 *gene was up-regulated in all the strains, while *ack1 *was up-regulated and *ack3 *down-regulated in 23K and LS 25 (Table 1). An illustration of the metabolic pathways with genes affected by the change of carbon source from glucose to ribose in *L. sakei *is shown in Figure 2.

As a consequence of the pentose-induced PKP, genes involved in PKP-metabolism of glucose, such as *gntZ*, *gntK *and *zwf*, were down-regulated (Table 1, Figure 2). The glycolytic pathway was clearly repressed, supporting previous findings [15,19]. Among these genes were *pfk *(0.5-1.1) encoding 6-phosphofructokinase (Pfk), and *fba *(0.7-1.1) coding for fructose-bisphosphate aldolase, both acting at the initial steps of glycolysis. In addition, *gpm3 *encoding one of the five phosphoglycerate mutases present in the 23K genome, acting in the lower part of glycolysis, was also down-regulated (0.7-0.9). MF1053 down-regulated *pyk *(0.7) encoding pyruvate kinase (Pyk) that competes for PEP with the PTS (Figure 2). Its activity results in the production of pyruvate and ATP, and it is of major importance in glycolysis and energy production in the cell. MF1053 also showed a stronger down-regulation of *pfk *than the other strains (Table 1). Similar to several other lactobacilli, *pfk *is transcribed together with *pyk *[43,44], and in many microorganisms the glycolytic flux depends on the activity of the two enzymes encoded from this operon [43,45]. At the protein level, we previously observed both Pfk and Pyk expressed at a lower level for all the three strains [19], however this was not confirmed at the level of gene expression for 23K and LS 25. We could also not confirm the lower protein expression of glyceraldehyde-3-phosphate dehydrogenase, phosphoglycerate kinase and enolase previously seen in LS 25 [19]. The latter three enzymes are encoded from the central glycolytic operon (*cggR-gap-pgk-tpi-eno*) together with triose-phosphate isomerase and the putative central glycolytic genes regulator (CggR) [46]. Besides the *cggR *gene being down-regulated in MF1053 and LS 25, no change in gene expression was seen of these central glycolytic genes. Thus at the transcription level it is not obvious that the LS 25 strain down-regulate the glycolytic pathway more efficiently than the other strains, as previously suggested [19].

Interestingly, all the strains showed an induction (1.4-2.3) of *mgsA *encoding methylglyoxal synthase, which catalyzes the conversion of dihydroxyacetone-phosphate to methylglyoxal (Figure 2). The presence of this gene is uncommon among LAB and so far a unique feature among the sequenced lactobacilli. The methylglyoxal pathway represents an energetically unfavourable bypass to the glycolysis. In *E. coli*, this bypass occurs as a response to phosphate starvation or uncontrolled carbohydrate metabolism, and enhanced ribose uptake was shown to lead to the accumulation of methylglyoxal [47,48]. As suggested by Chaillou et al. [7], such flexibility in the glycolytic process in *L. sakei *may reflect the requirement to deal with glucose starvation or to modulate carbon flux during co-metabolism of alternative carbon sources. Breakdown of methylglyoxal is important as it is toxic to the cells [49]. An induction of the *lsa1158 *gene contiguous with *mgsA *was seen for 23K and MF1053. This gene encodes a hypothetical protein, also suggested as a putative oxidoreductase, which may reduce methylglyoxal to lactaldehyde [7]. However, no induction of the *adhE *(*lsa0379*) gene encoding an iron-containing aldehyde dehydrogenase suggested to further reduce lactaldehyde to L-lactate [7] was seen. By CGH [32]*lsa1158 *and *adhE *were present in all the *L. sakei *strains investigated, whereas *mgsA *was lacking in some strains, indicating that the MgsA function is not vital.

### Pyruvate metabolism

Pyruvate is important in both glycolysis and PKP. It can be converted into lactate by the NAD-dependent L-lactate dehydrogenase, which regenerates NAD^+ ^and maintains the redox balance. This enzyme is encoded by the *ldhL *gene which was down-regulated (0.7-1.4) in all three strains, in accordance with previous findings [50], and the down-regulation was strongest for the LS 25 strain. At the protein level, only LS 25 showed a lower expression of this enzyme during growth on ribose [19]. Genes responsible for alternative fates of pyruvate (Figure 2) were highly induced in all the strains, however with some interesting strain variation (Table 1). The shift in pyruvate metabolism can benefit the bacteria by generating ATP, or by gaining NAD^+ ^for maintaining the redox balance and may lead to various end products in addition to lactate [51].

In all the strains, a strongly up-regulated (2.1-3.0) *pox1 *gene was observed, and in 23K an up-regulated *pox2 *(0.7), encoding pyruvate oxidases which under aerobic conditions convert pyruvate to acetyl-phosphate with hydrogen peroxide (H_2_O_2_) and CO_2 _as side products. Accumulation of peroxide ultimately leads to aerobic growth arrest [52]. H_2_O_2 _belongs to a group of compounds known as reactive oxygen species and reacts readily with metal ions to yield hydroxyl radicals that damage DNA, proteins and membranes [53]. Remarkable differences in redox activities exist among *Lactobacillus *species and *L. sakei *is among those extensively well equipped to cope with changing oxygen conditions, as well as dealing effectively with toxic oxygen byproducts [7]. 23K up-regulated *npr *(1.0) encoding NADH peroxidase which decomposes low concentrations of H_2_O_2 _to H_2_O and O_2_, and all the strains up-regulated the *sodA *gene (1.7-3.4) encoding a superoxide dismutase which produces hydrogen peroxide from superoxide (O_2_^-^). Various oxidoreductases showed an up-regulation in all the strains (Table 1), indicating the need for the bacterium to maintain its redox balance.

The *pdhABCD *gene cluster encoding components of the pyruvate dehydrogenase enzyme complex (PDC) which transforms pyruvate into acetyl-CoA and CO_2 _were among the strongly up-regulated (2.1-3.7) genes. The *eutD *gene encoding a phosphate acetyltransferase which further forms acetyl-phosphate from acetyl-CoA was also induced (1.0-2.0). Pyruvate can be transformed to acetolactate by acetolactate synthase and further to acetoin by acetolactate decarboxylase, before 2,3-butanediol may be formed by an acetoin recuctase (Figure 2). While the *budC *gene encoding the acetoin reductase showed a strong up-regulation in all three strains, the *als-aldB *operon was only strongly up-regulated in LS 25 (1.9). Pyruvate formate lyase produces acetyl-CoA and formate from pyruvate. Only in 23K, the *pflAB *genes encoding formate C-acetyltransferase and its activating enzyme involved in formate formation were strongly up-regulated (4.0 and 1.7, respectively). This strain was the only one to strongly induce L-lactate oxidase encoding genes which are responsible for conversion of lactate to acetate when oxygen is present (Table 1). In 23K and LS 25, the *ppdK *gene coding for the pyruvate phosphate dikinase involved in regenerating PEP, was induced, as was also *lsa0444 *encoding a putative malate dehydrogenase that catalyzes the conversion of malate into oxaloacetate using NAD^+ ^and vice versa (Table 1).

During growth on ribose, *L. sakei *was shown to require thiamine (vitamine B1) [15]. The E1 component subunit α of the PDC, as well as Pox and Xpk, require thiamine pyrophosphate, the active form of thiamine, as a coenzyme [54]. This could explain the induction of the *thiMDE *operon and *lsa0055 *in LS 25, as well as *lsa0980 *in 23K, encoding enzymes involved in thiamine uptake and biosynthesis (Table 1). The up-regulation of *lsa1664 *(1.1-1.6) encoding a putative dihydrofolate reductase involved in biosynthesis of riboflavin (vitamin B2) in all the strains could indicate a requirement for flavin nucleotides as enzyme cofactors. Riboflavin is the precursor for flavin mononucleotide (FMN) and flavin adenine dinucleotide (FAD) redox cofactors in flavoproteins, and the E3 component of PDC as well as glycerol-3-phosphate dehydrogenase encoded from the up-regulated *glpD*, are among enzymes requiring FAD. Another cofactor which seems to be important during growth on ribose is lipoate, essential of the E2 component of the PDC. An up-regulation of *lplA *(1.0 - 1.6) encoding lipoate-protein ligase, which facilitates attachment of the lipoyl moiety to metabolic enzyme complexes, was seen in all the strains, allowing the bacterium to scavenge extracellular lipoate [55,56].

### Nucleoside catabolism

The *L. sakei *genome contains a multiplicity of catabolic genes involved in exogenous nucleoside salvage pathways, and the bacterium has been shown to catabolize inosine and adenosine for energy [7]. Three *iunH *genes are present in the 23K genome, which encode inosine-uridine preferring nucleoside hydrolases responsible for conversion of inosine to ribose and purine base. The *iunH1 *gene was up-regulated in all the strains when grown on ribose (1.8-2.6), as was also the *iunH2 *gene in 23K (1.2). The *deoC *gene encodes a deoxyribose-phosphate aldolase, and is located in an operon structure preceding the genes *deoB*, *deoD*, *lsa0798*, *lsa0799*, *deoR *and *pdp *which encode phosphopentomutase, purine nucleoside phosphorylase, pyrimidine-specific nucleoside symporter, a putative purine transport protein, the deoxyribonucleoside synthesis operon transcriptional regulator (DeoR), and a pyrimidine-nucleoside phosphorylase, respectively. The complete operon was induced in all the strains, except for *pdp *only induced in 23K (Table 1). The phosphorylases catalyze cleavage of ribonucleosides and deoxyribonucleosides to the free base pluss ribose-1-phosphate or deoxyribose-1-phosphate. The bases are further utilized in nucleotide synthesis or as nitrogen sources. The pentomutase converts ribose-1-phosphate or deoxyribose-1-phosphate to ribose-5-phosphate or deoxyribose-5-phosphate, respectively, which can be cleaved by the aldolase to glyceraldehyde-3-phosphate and acetaldehyde. Glyceraldehyde-3-phosphate enters the glycolysis, while a putative iron containing alcohol dehydrogenase, encoded by *lsa0258 *up-regulated in all the strains (0.5-1.6), could further reduce acetaldehyde to ethanol (Figure 2). The obvious induced nucleoside catabolism at the level of gene expression was not seen by proteomic analysis [19].

### Genes involved in glycerol/glycerolipid/fatty acid metabolism

During growth on ribose, a strong induction of the *glpKDF *operon encoding glycerol kinase (GlpK), glycerol-3-phosphate dehydrogenase (GlpD), and glycerol uptake facilitator protein was observed (Table 1), which is in correlation with the over-expression of GlpD and GlpK seen by proteomic analysis [19]. GlpD is FADH_2 _linked and converts glycerol-3-phosphate to dihydroxyacetone-phosphate. An over-expression of GlpD was also reported when *L. sakei *was exposed to low temperature [57]. A *glpD *mutant showed enhanced survival at low temperature, and it was suggested that this was a result of the glycerol metabolism being redirected into phosphatidic acid synthesis which leads to membrane phospholipid biosynthesis [57]. Nevertheless, a down-regulation was observed of the *lsa1493 *gene (0.6-0.9) encoding a putative diacylglycerol kinase involved in the synthesis of phosphatidic acid, and of *cfa *(1.3-1.4) encoding cyclopropane-fatty-acyl-phospholipid synthase directly linked to modifications in the bacterial membrane fatty acid composition that reduce membrane fluidity and helps cells adapt to their environment [58]. Interestingly, LS 25 up-regulated several genes (LSA0812-0823), including *accD *and *accA *encoding the α- and ß-subunits of the multi-subunit acetyl-CoA carboxylase (Table 1). This is a biotin-dependent enzyme that catalyzes the irreversible carboxylation of acetyl-CoA to produce malonyl-CoA, an essential intermediate in fatty acid biosynthesis. In *B. subtilis*, the malonyl-CoA relieves repression of the *fab *genes [59]. We observed that also *acpP*, *fabZ1*, *fabH*, *fabD *and *fabI *(Table 1) encoding enzymes involved in fatty acid biosynthesis were induced in LS 25. The altered flux to malonyl-CoA may be a result of the decreased glycolytic rate. MF1053, on the other hand, showed a down-regulation of several genes in the same gene cluster. A higher level of acetate is produced when the bacterium utilizes ribose, and acetate lowers the pH and has a higher antimicrobial effect than lactate. Changes in the phospholipid composition could be a response to changes in intracellular pH. Protons need to be expelled at a higher rate when the pH drops. The LS 25 strain which showed faster growth rates than the other strains [9], was the only strain to up-regulate the F_0_F_1 _ATP synthase (Table 1), which at the expense of ATP expels protons during low pH.

### Regulation mechanisms

Little is known about the regulation of catabolic pathways in *L. sakei*. Starting from ribose uptake, the *rbs *operon may be both relieved from repression and ribose induced. Presumably, a dual regulation of this operon by two opposite mechanisms, substrate induction by ribose and CCR by glucose may occur in *L. sakei*. The *ccpA *gene was not regulated, consistent with this gene commonly showing constitutive expression in lactobacilli [42,60]. The local repressor RbsR is homologous with CcpA, both belonging to the same LacI/GalR family of transcriptional regulators. RbsR was proposed to bind a *cre*-like consensus sequence located close to a putative CcpA *cre *site, both preceding *rbsU *[28]. RbsR in the Gram-positive soil bacterium *Corynebacterium glutamicum *was shown to bind a *cre*-like sequence, and using microarrays, the transcription of no other genes but the *rbs *operon was affected positively in an *rbsR *deletion mutant. It was concluded that RbsR influences the expression of only the *rbs *operon [61]. Similarily, in the *L. sakei *sequence, no other candidate members of RbsR regulation could be found [28]. However, experiments are needed to confirm RbsR binding in

*L. sakei*. In *Bacillus subtilis*, RbsR represent a novel interaction partner of P-Ser-HPr in a similar fashion to CcpA [62]. The P-Ser-HPr interaction is possible also in *L. sakei *as the bacterium exhibits HPr-kinase/phosphatase activity.

A putative *cre *site is present in the promoter of *lsa0254 *encoding the second ribokinase (Table 2), and this gene is preceeded by the opposite oriented gene *lsa0253 *encoding a transcriptional regulator with a sugar binding domain which belongs to the GntR family. This family of transcriptional regulators, as well as the LacI family which RbsR and CcpA belong to, are among the families to which regulators involved in carbohydrate uptake or metabolism usually belong [63]. The GntR-type regulator could possibly be involved in regulating the expression of the second ribokinase, or of the inosine-uridine preferring nucleoside hydrolase encoding *iunH1 *gene which is located further upstream of *lsa0254*. *C. glutamicum *possesses an operon encoding a ribokinase, a uridine transporter, and a uridine-preferring nucleoside hydrolase which is co-controlled by a local repressor together with the RbsR repressor of the *rbs *operon [60,61,64]. It is possible that such co-control could exist also in *L. sakei*. Ribose as well as nucleosides are products of the degradation of organic materials such as DNA, RNA and ATP. The simultaneous expression of the *rbs *and *deo *operons as well as the other genes involved in ribose and nucleoside catabolism (Figure 2) allows the bacterium to access the different substrates simultaneously and use both ribose as well as nucleosides as carbon and energy source. DeoR shows 51% identity to the *B. subtilis *DeoR repressor protein [65,66]. Genes encoding deoxyribose-phosphate aldolase, nucleoside uptake protein and pyrimidine nucleoside phosphorylase in *B. subtilis *are organized in a *dra-nupC-pdp *operon followed by *deoR*, and ribose was shown to release DeoR from DNA binding and thus repression of the operon genes are alleviated [65-67]. The *B. subtilis *pentomutase and purine-nucleoside phosphorylase are encoded from a *drm-pupG *operon which is not negatively regulated by DeoR, though both operons are subject to CcpA mediated CCR [65,66,68]. As a *cre *site is found preceding the *L. sakei deoC *(Table 2), the operon could be regulated by CcpA as well. It is interesting that *deoR *is the only strongly induced transcriptional regulator gene in all three strains, and the encoded regulator has sigma (σ) factor activity. We can only speculate whether it could function as activator of transcription on some of the regulated genes in this study.

Expression of the Xpk encoding gene of *Lactobacillus pentosus *was reported to be induced by sugars fermented through the PKP and repressed by glucose mediated by CcpA [69]. Indeed, the *cre *site overlapping ATG start codon of *L. sakei xpk *(Table 2) indicates relief of CcpA-mediated CCR during growth on ribose. Also for several genes involved in alternative fates of pyruvate, putative *cre *sites were present (Table 2).

Several genes and operons involved in transport and metabolism of various carbohydrates such as mannose, galactose, fructose, lactose, cellobiose, N-acetylglucosamine, including putative sugar kinases and PTSs, were induced during growth on ribose (Table 1), and as shown in Table 2, putative *cre *sites are located in the promoter region of many of these up-regulated genes and operons. 23K showed an up-regulation of genes involved in the arginine deiminase pathway, and 23K and LS 25 showed an up-regulated threonine deaminase (Table 1). The *arcA *and *tdcB *both have putative *cre *sites in their promoter regions (Table 2). Thus ribose seems to induce a global regulation of carbon metabolism in *L. sakei*.

A putative *cre *site precedes the *glp *operon (Table 2), suggesting regulation mediated by CcpA. However, regulation of the *L. sakei *GlpK may also occur by an inducer exclusion-based CcpA-independent CCR mechanism as described in enterococci and *B. subtilis *[70,71], and as previously suggested by Stentz et al. [15]. By this mechanism, glycerol metabolism is regulated by PEP-dependent, EI- and HPr-catalyzed phosphorylation of GlpK in response to the presence or absence of a PTS substrate. In the absence of a PTS sugar, GlpK is phosphorylated by P-His-HPr at a conserved histidyl residue, forming the active P-GlpK form, whereas during growth on a PTS sugar, phosphoryl transfer flux through the PTS is high, concentration of P-His-HPr is low, and GlpK is present in a less active dephospho form [20,70,71]. This conserved histidyl residue (His232) is present in *L. sakei *GlpK [20], and Stentz et al. [15] reported that whereas *L. sakei *can grow poorly on glycerol, this growth was abolished in *ptsI *mutants.

### Mannose-PTS

As mentioned in the introduction, the PTS plays a central role, in both the uptake of a number of carbohydrates and regulatory mechanisms [20-22]. Encoding the general components, *ptsH *showed an up-regulation in MF1053 and LS 25 (1.2 and 0.9, respectively), while all the strains up-regulated *ptsI *(0.8-1.7). The *manLMN *operon encoding the EII^man ^complex was surprisingly strongly up-regulated during growth on ribose in all the strains (Table 1). By proteomic analysis, no regulation of the PTS enzymes was seen [19]. The expression of HPr and EI in *L. sakei *during growth on glucose or ribose was previously suggested to be constitutive [14], and in other lactobacilli, the EII^man ^complex was reported to be consistently highly expressed, regardless of carbohydrate source [72-74]. Notably, PEP-dependent phosphorylation of PTS sugars has been detected in ribose-grown cells, indicating that the EII^man ^complex is active, and since no transport and phosphorylation via EII^man ^occurs, the complex is phosphorylated, while it is unphosphorylated in the presence of the substrates of the EII^man ^complex [8,73]. The stimulating effect exerted by small amounts of glucose on ribose uptake in *L. sakei*, which has also been reported in other lactobacilli [74,75], was suggested to be caused by dephosphorylation of the PTS proteins in the presence of glucose, as a *ptsI *mutant lacking EI, as well as P-His-HPr, was shown to enhance ribose uptake [15,16,76]. Stentz et al. [15] observed that a *L. sakei *mutant (strain RV52) resistant to 2 deoxy-D-glucose, a glucose toxic analog transported by EII^man^, and thus assumed to be affected in the EII^man^, did not show the same enhanced uptake [15]. It was concluded that EII^man ^is not involved in the PTS-mediated regulation of ribose metabolism in *L. sakei*. The mutation was though not reported verified by sequencing [15], and other mutations could be responsible for the observed phenotype. The *L. sakei *EIIAB^man^, EIIC^man ^and EIID^man ^show 72, 81, and 82% identity, respectively, with the same enzymes in *L. casei*, in which mutations rendering the EII^man ^complex inactive were shown to derepress *rbs *genes, resulting in a loss of the preferential use of glucose over ribose [75]. Furthermore, in *L. pentosus*, EII^man ^was shown to provide a strong signal to the CcpA-dependent repression pathway [73]. The *hprK *gene encoding HPrK/P which controls the phosphorylation state of HPr was strongly up-regulated (1.2-2.0) in all three strains. HPrK/P dephosphorylates P-Ser-HPr when the concentration of glycolytic intermediates drop, which is likely the situation during growth on ribose [20,22,24].

Numerous genes encoding hypothetical proteins with unknown function were also found to be differentially expressed (Table 1), as well as several other genes belonging to various functional categories. For most of these, their direct connection with ribose metabolism is unknown, and is likely an indirect effect.

## Conclusions

The ability to ferment meat and fish is related to the capacity of the bacterium to rapidly take up the available carbohydrates and other components for growth. The importance of this process, especially to the meat industry, stimulates research aimed at understanding the mechanisms for transport and metabolism of these compounds, with the ultimate goal to be able to select improved strains. Genome-wide transcriptome analyses with DNA microarrays efficiently allowed the identification of genes differentially expressed between growth on the two carbohydrates which *L. sakei *can utilize from these substrates. Moreover, microarrays were a powerful tool to increase the understanding of the bacterium's primary metabolism and revealed a global regulatory mechanism. In summary, the ribose uptake and catabolic machinery is highly regulated at the transcription level, and it is closely linked with catabolism of nucleosides. A global regulation mechanism seems to permit a fine tuning of the expression of enzymes that control efficient exploitation of available carbon sources.

## Abbreviations

PKP: phosphoketolase pathway; PEP: phosphoenolpyruvate; PTS: PEP-dependent carbohydrate phosphotransferase system; CCR: carbon catabolite repression; *cre*: catabolite responsive element; RbsK: ribokinase; RbsD: D-Ribose pyranase; Xpk: xylulose-5-phosphate phosphoketolase; Ack: Acetate kinase, Pfk: 6-phosphofructokinase; Pyk: pyruvate kinase; PDC: pyruvate dehydrogenase complex; GlpD: glycerol-3-phosphate dehydrogenase; GlpK: glycerol kinase; EII: enzyme II; EI: enzyme I; HPr: histidine protein; HPrK/P: HPr kinase/phosphatase; DeoR: deoxyribonucleoside synthesis operon transcriptional regulator.

## Competing interests

The authors declare that they have no competing interests.

## Authors' contributions

AM participated in the study design, conducted the experimental work, analyzed and interpreted data, and wrote the manuscript. LS conducted the statistical analysis. KN and LA conceived the study, participated in the study design process and reviewed the manuscript. All authors read and approved the final manuscript.

## Supplementary Material

Additional file 1 **Table S3**. **Primer and probe sets used for qRT-PCR**. Presents the primer and probe sets used for validation of microarray data by qRT-PCR analysis. **Table S4**. **Comparison of microarray data with qRT-PCR results of *L***. ***sakei *strain LS 25 grown on ribose compared with glucose**. Presents gene regulation values (log_2_) from the qRT-PCR analysis in comparison with microarray data. Click here for file

## References

[B1] Hammes WP Bantleon A Min S Lactic acid bacteria in meat fermentation FEMS Microbiol Rev 1990 87 165 174

[B2] Hammes WP Hertel C New developments in meat starter cultures Meat Science 1998 49 125 138 22060705

[B3] Bredholt S Nesbakken T Holck A Protective cultures inhibit growth of *Listeria monocytogenes *and *Escherichia coli *O157:H7 in cooked, sliced, vacuum- and gas-packaged meat Int J Food Microbiol 1999 53 43 52 10.1016/s0168-1605(99)00147-6 10598113

[B4] Bredholt S Nesbakken T Holck A Industrial application of an antilisterial strain of *Lactobacillus sakei *as a protective culture and its effect on the sensory acceptability of cooked, sliced, vacuum-packaged meats Int J Food Microbiol 2001 66 191 196 10.1016/s0168-1605(00)00519-5 11428578

[B5] Katikou P Georgantelis D Paleologos EK Ambrosiadis I Kontominas MG Relation of biogenic amines' formation with microbiological and sensory attributes in *Lactobacillus*-inoculated vacuum-packed rainbow trout (*Oncorhynchus mykiss*) fillets J Agric Food Chem 2006 54 4277 4283 10.1021/jf0602121 16756357

[B6] Vermeiren L Devlieghere F Debevere J Evaluation of meat born lactic acid bacteria as protective cultures for biopreservation of cooked meat products Int J Food Microbiol 2004 96 149 164 10.1016/j.ijfoodmicro.2004.03.016 15364469

[B7] Chaillou S Champomier-Vergès MC Cornet M Crutz-Le Coq AM Dudez AM Martin V Beaufils S Darbon-Rongere E Bossy R Loux V Zagorec M The complete genome sequence of the meat-borne lactic acid bacterium *Lactobacillus sakei *23 K Nat Biotechnol 2005 23 1527 1533 10.1038/nbt1160 16273110

[B8] Lauret R Morel-Deville F Berthier F Champomier-Vergès M Postma P Ehrlich SD Zagorec M Carbohydrate utilization in *Lactobacillus sake* Appl Environ Microbiol 1996 62 1922 1927 10.1128/aem.62.6.1922-1927.1996 PMC1388869 16535331

[B9] McLeod A Nyquist OL Snipen L Naterstad K Axelsson L Diversity of *Lactobacillus sakei *strains investigated by phenotypic and genotypic methods Syst Appl Microbiol 2008 31 393 403 10.1016/j.syapm.2008.06.002 18678454

[B10] Chiaramonte F Blugeon S Chaillou S Langella P Zagorec M Behavior of the meat-borne bacterium *Lactobacillus sakei *during its transit through the gastrointestinal tracts of axenic and conventional mice Appl Environ Microbiol 2009 75 4498 4505 10.1128/AEM.02868-08 PMC2704804 19447958

[B11] Dal Bello F Walter J Hammes WP Hertel C Increased complexity of the species composition of lactic acid bacteria in human feces revealed by alternative incubation condition Microb Ecol 2003 45 455 463 10.1007/s00248-003-2001-z 12704557

[B12] Walker A Cerdeno-Tarraga A Bentley S Faecal matters Nat Rev Microbiol 2006 4 572 573 10.1038/nrmicro1483 16888877

[B13] Chiaramonte F Anglade P Baraige F Gratadoux JJ Langella P Champomier-Vergès MC Zagorec M Analysis of *Lactobacillus sakei *mutants selected after adaptation to the gastrointestinal tract of axenic mice Appl Environ Microbiol 2010 76 2932 2939 10.1128/AEM.02451-09 PMC2863443 20208026

[B14] Stentz R Lauret R Ehrlich SD Morel-Deville F Zagorec M Molecular cloning and analysis of the *ptsHI *operon in *Lactobacillus sake* Appl Environ Microbiol 1997 63 2111 2116 10.1128/aem.63.6.2111-2116.1997 PMC168499 9172326

[B15] Stentz R Cornet M Chaillou S Zagorec M Adaption of *Lactobacillus sakei *to meat: a new regulatory mechanism of ribose utilization? INRA, EDP Sciences 2001 81 131 138

[B16] Stentz R Zagorec M Ribose utilization in *Lactobacillus sakei*: analysis of the regulation of the *rbs *operon and putative involvement of a new transporter J Mol Microbiol Biotechnol 1999 1 165 173 10941799

[B17] Torriani S Clementi F Vancanneyt M Hoste B Dellaglio F Kersters K Differentiation of *Lactobacillus plantarum*, *L. pentosus *and *L. paraplantarum *species by RAPD-PCR and AFLP Syst Appl Microbiol 2001 24 554 560 10.1078/0723-2020-00071 11876363

[B18] Claesson MJ van Sinderen D O'Toole PW The genus *Lactobacillus *- a genomic basis for understanding its diversity FEMS Microbiol Lett 2007 269 22 28 10.1111/j.1574-6968.2006.00596.x 17343688

[B19] McLeod A Zagorec M Champomier-Vergès MC Naterstad K Axelsson L Primary metabolism in *Lactobacillus sakei *food isolates by proteomic analysis BMC Microbiol 2010 10 120 10.1186/1471-2180-10-120 PMC2873491 20412581

[B20] Deutscher J Francke C Postma PW How phosphotransferase system-related protein phosphorylation regulates carbohydrate metabolism in bacteria Microbiol Mol Biol Rev 2006 70 939 1031 10.1128/MMBR.00024-06 PMC1698508 17158705

[B21] Stulke J Hillen W Carbon catabolite repression in bacteria Curr Opin Microbiol 1999 2 195 201 10.1016/S1369-5274(99)80034-4 10322165

[B22] Titgemeyer F Hillen W Global control of sugar metabolism: a gram-positive solution Antonie Van Leeuwenhoek 2002 82 59 71 12369205

[B23] Fujita Y Carbon catabolite control of the metabolic network in *Bacillus subtilis* Biosci Biotechnol Biochem 2009 73 245 259 10.1271/bbb.80479 19202299

[B24] Schumacher MA Allen GS Diel M Seidel G Hillen W Brennan RG Structural basis for allosteric control of the transcription regulator CcpA by the phosphoprotein HPr-Ser46-P Cell 2004 118 731 741 10.1016/j.cell.2004.08.027 15369672

[B25] Obst M Hehn R Vogel RF Hammes WP Lactose metabolism in *Lactobacillus curvatus *and *Lactobacillus sake* FEMS Microbiol Lett 1992 97 209 214

[B26] Montel MC Champomier MC Arginine catabolism in *Lactobacillus sake *isolated from meat Appl Environ Microbiol 1987 53 2683 2685 10.1128/aem.53.11.2683-2685.1987 PMC204175 3426226

[B27] Zuniga M Champomier-Vergès M Zagorec M Pérez-Martinez G Structural and functional analysis of the gene cluster encoding the enzymes of the arginine deiminase pathway of *Lactobacillus sake* J Bacteriol 1998 180 4154 4159 10.1128/jb.180.16.4154-4159.1998 PMC107411 9696763

[B28] Rodionov DA Mironov AA Gelfand MS Transcriptional regulation of pentose utilisation systems in the *Bacillus/Clostridium *group of bacteria FEMS Microbiol Lett 2001 205 305 314 10.1111/j.1574-6968.2001.tb10965.x 11750820

[B29] Berthier F Zagorec M Champomier-Vergès MC Ehrlich SD Morel-Deville F Efficient transformation of *Lactobacillus sake *by electroporation Microbiol 1996 142 1273 1279 10.1099/13500872-142-5-1273 33725790

[B30] Hagen BF Næs H Holck AL Meat starters have individual requirements for Mn2+ Meat Science 2000 55 161 168 10.1016/s0309-1740(99)00138-2 22061081

[B31] Møretrø T Hagen BF Axelsson L A new, completely defined medium for meat lactobacilli J Appl Microbiol 1998 85 715 722

[B32] Nyquist OL McLeod A Brede DA Snipen L Nes IF Comparative genomics of *Lactobacillus sakei *with emphasis on strains from meat Mol Genet Genomics 2011 285 297 311 10.1007/s00438-011-0608-1 21369871

[B33] Rud I Naterstad K Bongers RS Molenaar D Kleerebezem M Axelsson L Functional analysis of the role of CggR (central glycolytic gene regulator) in *Lactobacillus plantarum *by transcriptome analysis Microbial Biotechnology 2011 4 345 356 10.1111/j.1751-7915.2010.00223.x PMC3818993 21375718

[B34] Vebø HC Solheim M Snipen L Nes IF Brede DA Comparative genomic analysis of pathogenic and probiotic *Enterococcus faecalis *isolates, and their transcriptional responses to growth in human urine PLoS One 2010 5 e12489 10.1371/journal.pone.0012489 PMC2930860 20824220

[B35] Smyth GK Speed T Normalization of cDNA microarray data Methods 2003 31 265 273 10.1016/s1046-2023(03)00155-5 14597310

[B36] Smyth GK Linear models and empirical bayes methods for assessing differential expression in microarray experiments Stat Appl Genet Mol Biol 2004 3 Article3 10.2202/1544-6115.1027 16646809

[B37] Smyth GK Michaud J Scott HS Use of within-array replicate spots for assessing differential expression in microarray experiments Bioinformatics 2005 21 2067 2075 10.1093/bioinformatics/bti270 15657102

[B38] Rode TM Møretrø T Langsrud S Langsrud O Vogt G Holck A Responses of *Staphylococcus aureus *exposed to HCl and organic acid stress Can J Microbiol 2010 56 777 792 10.1139/w10-057 20921988

[B39] Weickert MJ Chambliss GH Site-directed mutagenesis of a catabolite repression operator sequence in *Bacillus subtilis* Proc Natl Acad Sci USA 1990 87 6238 6242 10.1073/pnas.87.16.6238 PMC54508 2117276

[B40] Champomier-Vergès MC Chaillou S Cornet M Zagorec M Erratum to "*Lactobacillus sakei*: recent developments and future prospects" Res Microbiol 2002 153 115 123 10.1016/s0923-2508(01)01296-7 11900264

[B41] Lorquet F Goffin P Muscariello L Baudry JB Ladero V Sacco M Kleerebezem M Hols P Characterization and functional analysis of the *poxB *gene, which encodes pyruvate oxidase in *Lactobacillus plantarum* J Bacteriol 2004 186 3749 3759 10.1128/JB.186.12.3749-3759.2004 PMC419957 15175288

[B42] Muscariello L Marasco R De Felice M Sacco M The functional *ccpA *gene is required for carbon catabolite repression in *Lactobacillus plantarum* Appl Environ Microbiol 2001 67 2903 2907 10.1128/AEM.67.7.2903-2907.2001 PMC92959 11425700

[B43] Branny P De La Torre F Garel JR Cloning, sequencing, and expression in *Escherichia coli *of the gene coding for phosphofructokinase in *Lactobacillus bulgaricus* J Bacteriol 1993 175 5344 5349 10.1128/jb.175.17.5344-5349.1993 PMC206588 8366023

[B44] Viana R Perez-Martinez G Deutscher J Monedero V The glycolytic genes *pfk *and *pyk *from *Lactobacillus casei *are induced by sugars transported by the phosphoenolpyruvate:sugar phosphotransferase system and repressed by CcpA Arch Microbiol 2005 183 385 393 10.1007/s00203-005-0003-6 16075200

[B45] Kandler O Carbohydrate metabolism in lactic acid bacteria Antonie Van Leeuwenhoek 1983 49 209 224 10.1007/BF00399499 6354079

[B46] Naterstad K Rud I Kvam I Axelsson L Characterisation of the *gap *operon from *Lactobacillus plantarum *and *Lactobacillus sakei* Curr Microbiol 2007 54 180 185 10.1007/s00284-006-0013-x 17294332

[B47] Kim I Kim E Yoo S Shin D Min B Song J Park C Ribose utilization with an excess of mutarotase causes cell death due to accumulation of methylglyoxal J Bacteriol 2004 186 7229 7235 10.1128/JB.186.21.7229-7235.2004 PMC523224 15489434

[B48] Weber J Kayser A Rinas U Metabolic flux analysis of *Escherichia coli *in glucose-limited continuous culture. II. Dynamic response to famine and feast, activation of the methylglyoxal pathway and oscillatory behaviour Microbiology 2005 151 707 716 10.1099/mic.0.27482-0 15758217

[B49] Totemeyer S Booth NA Nichols WW Dunbar B Booth IR From famine to feast: the role of methylglyoxal production in *Escherichia coli* Mol Microbiol 1998 27 553 562 10.1046/j.1365-2958.1998.00700.x 9489667

[B50] Malleret C Lauret R Ehrlich SD Morel-Deville F Zagorec M Disruption of the sole *ldhL *gene in *Lactobacillus sakei *prevents the production of both L- and D-lactate Microbiology 1998 144 3327 3333 10.1099/00221287-144-12-3327 9884224

[B51] Axelsson L Salminen S, von Wright A, Ouwehand A Lactic acid bacteria: classification and physiology Lactic acid bacteria: microbiological and functional aspects 2004 Third revised and expanded New York, USA: Marcel Dekker, Inc./CRC Press 1 66

[B52] Condon S Responses of lactic acid bacteria to oxygen FEMS Microbiol Rev 1987 46 269 280

[B53] Fridovich I The biology of oxygen radicals Science 1978 201 875 880 10.1126/science.210504 210504

[B54] Rodionov DA Vitreschak AG Mironov AA Gelfand MS Comparative genomics of thiamin biosynthesis in procaryotes. New genes and regulatory mechanisms J Biol Chem 2002 277 48949 48959 10.1074/jbc.M208965200 12376536

[B55] Jordan A Reichard P Ribonucleotide reductases Annu Rev Biochem 1998 67 71 98 10.1146/annurev.biochem.67.1.71 9759483

[B56] Keeney KM Stuckey JA O'Riordan MX LplA1-dependent utilization of host lipoyl peptides enables *Listeria *cytosolic growth and virulence Mol Microbiol 2007 66 758 770 10.1111/j.1365-2958.2007.05956.x PMC2367003 17908209

[B57] Marceau A Zagorec M Chaillou S Mera T Champomier-Vergès MC Evidence for involvement of at least six proteins in adaptation of *Lactobacillus sakei *to cold temperatures and addition of NaCl Appl Environ Microbiol 2004 70 7260 7268 10.1128/AEM.70.12.7260-7268.2004 PMC535173 15574925

[B58] Grogan DW Cronan JE Jr Cyclopropane ring formation in membrane lipids of bacteria Microbiol Mol Biol Rev 1997 61 429 441 10.1128/mmbr.61.4.429-441.1997 PMC232619 9409147

[B59] Schujman GE Guerin M Buschiazzo A Schaeffer F Llarrull LI Reh G Vila AJ Alzari PM de Mendoza D Structural basis of lipid biosynthesis regulation in Gram-positive bacteria Embo J 2006 25 4074 4083 10.1038/sj.emboj.7601284 PMC1560364 16932747

[B60] Mahr K Hillen W Titgemeyer F Carbon catabolite repression in *Lactobacillus pentosus*: analysis of the *ccpA *region Appl Environ Microbiol 2000 66 277 283 10.1128/aem.66.1.277-283.2000 PMC91818 10618236

[B61] Nentwich SS Brinkrolf K Gaigalat L Huser AT Rey DA Mohrbach T Marin K Puhler A Tauch A Kalinowski J Characterization of the LacI-type transcriptional repressor RbsR controlling ribose transport in *Corynebacterium glutamicum *ATCC 13032 Microbiology 2009 155 150 164 10.1099/mic.0.020388-0 19118356

[B62] Muller W Horstmann N Hillen W Sticht H The transcription regulator RbsR represents a novel interaction partner of the phosphoprotein HPr-Ser46-P in *Bacillus subtilis* Febs J 2006 273 1251 1261 10.1111/j.1742-4658.2006.05148.x 16519689

[B63] Perez-Rueda E Collado-Vides J The repertoire of DNA-binding transcriptional regulators in *Escherichia coli *K-12 Nucleic Acids Res 2000 28 1838 1847 10.1093/nar/28.8.1838 PMC102813 10734204

[B64] Brinkrolf K Ploger S Solle S Brune I Nentwich SS Huser AT Kalinowski J Puhler A Tauch A The LacI/GalR family transcriptional regulator UriR negatively controls uridine utilization of *Corynebacterium glutamicum *by binding to catabolite-responsive element (*cre*)-like sequences Microbiology 2008 154 1068 1081 10.1099/mic.0.2007/014001-0 18375800

[B65] Saxild HH Andersen LN Hammer K *dra-nupC-pdp *operon of *Bacillus subtilis*: nucleotide sequence, induction by deoxyribonucleosides, and transcriptional regulation by the *deoR*-encoded DeoR repressor protein J Bacteriol 1996 178 424 434 10.1128/jb.178.2.424-434.1996 PMC177674 8550462

[B66] Zeng X Saxild HH Identification and characterization of a DeoR-specific operator sequence essential for induction of *dra-nupC-pdp *operon expression in *Bacillus subtilis* J Bacteriol 1999 181 1719 1727 10.1128/jb.181.6.1719-1727.1999 PMC93568 10074062

[B67] Zeng X Saxild HH Switzer RL Purification and characterization of the DeoR repressor of *Bacillus subtilis* J Bacteriol 2000 182 1916 1922 10.1128/jb.182.7.1916-1922.2000 PMC101877 10714997

[B68] Schuch R Garibian A Saxild HH Piggot PJ Nygaard P Nucleosides as a carbon source in *Bacillus subtilis*: characterization of the *drm-pupG *operon Microbiology 1999 145 2957 2966 10.1099/00221287-145-10-2957 10537218

[B69] Posthuma CC Bader R Engelmann R Postma PW Hengstenberg W Pouwels PH Expression of the xylulose 5-phosphate phosphoketolase gene, *xpkA*, from *Lactobacillus pentosus *MD363 is induced by sugars that are fermented via the phosphoketolase pathway and is repressed by glucose mediated by CcpA and the mannose phosphoenolpyruvate phosphotransferase system Appl Environ Microbiol 2002 68 831 837 10.1128/AEM.68.2.831-837.2002 PMC126734 11823225

[B70] Charrier V Buckley E Parsonage D Galinier A Darbon E Jaquinod M Forest E Deutscher J Claiborne A Cloning and sequencing of two enterococcal *glpK *genes and regulation of the encoded glycerol kinases by phosphoenolpyruvate-dependent, phosphotransferase system-catalyzed phosphorylation of a single histidyl residue J Biol Chem 1997 272 14166 14174 10.1074/jbc.272.22.14166 9162046

[B71] Darbon E Servant P Poncet S Deutscher J Antitermination by GlpP, catabolite repression via CcpA and inducer exclusion triggered by P-GlpK dephosphorylation control *Bacillus subtilis glpFK *expression Mol Microbiol 2002 43 1039 1052 10.1046/j.1365-2958.2002.02800.x 11929549

[B72] Barrangou R Azcarate-Peril MA Duong T Conners SB Kelly RM Klaenhammer TR Global analysis of carbohydrate utilization by *Lactobacillus acidophilus *using cDNA microarrays Proc Natl Acad Sci USA 2006 103 3816 3821 10.1073/pnas.0511287103 PMC1533782 16505367

[B73] Chaillou S Postma PW Pouwels PH Contribution of the phosphoenolpyruvate:mannose phosphotransferase system to carbon catabolite repression in *Lactobacillus pentosus* Microbiology 2001 147 671 679 10.1099/00221287-147-3-671 11238974

[B74] Veyrat A Gosalbes MJ Perez-Martinez G *Lactobacillus curvatus *has a glucose transport system homologous to the mannose family of phosphoenolpyruvate-dependent phosphotransferase systems Microbiology 1996 142 3469 3477 10.1099/13500872-142-12-3469 9004509

[B75] Veyrat A Monedero V Perez-Martinez G Glucose transport by the phosphoenolpyruvate:mannose phosphotransferase system in *Lactobacillus casei *ATCC 393 and its role in carbon catabolite repression Microbiology 1994 140 1141 1149 10.1099/13500872-140-5-1141 8025679

[B76] Viana R Monedero V Dossonnet V Vadeboncoeur C Perez-Martinez G Deutscher J Enzyme I and HPr from *Lactobacillus casei*: their role in sugar transport, carbon catabolite repression and inducer exclusion Mol Microbiol 2000 36 570 584 10.1046/j.1365-2958.2000.01862.x 10844647

