# Supplementary material for: Global transcriptome response in Lactobacillus sakei during growth on ribose
Source: BMC Microbiol. 2011 Jun 24;11:145. doi: 10.1186/1471-2180-11-145 (PMC3146418; doi:10.1186/1471-2180-11-145)
Supplement: Additional file 1 — Table S3. Primer and probe sets used for qRT-PCR. Presents the primer and probe sets used for validation of microarray data by qRT-PCR analysis. Table S4. Comparison of microarray data with qRT-PCR results of L. sakei strain LS 25 grown on ribose compared with glucose. Presents gene regulation values (log2) from the qRT-PCR analysis in comparison with microarray data. [file 1471-2180-11-145-S1.PDF]

Table S3. Primer and probe sets used for qRT-PCR.

Target gene	Primer and probe name	Sequence[a] (5'→3')
gyrA (LSA0006)	gyrA-F	TGGCGGCCGTGGAAT
	gyrA-R	ATTAAGTGTTCGATGAAATCGTCATC
	gyrA-Taq	CAAGGGATGGGTGTTC
lsa0254 (LSA0254)	lsa0254-F	TTTAGTTAACCAAGGAATTCGTCATG
	lsa0254-R	CTGTTTGACTTTCATCTGCACATAGA
	lsa0254-Taq	TTTTATCTCGCTTGGCGCACACGG
manL (LSA0449)	manL-F	GAAGCATACGCATCACGTTTATCT
	manL-R	CACCAGCCTTAGCTTCATCAATAAT
	manL-Taq	TGACATCTGCTCATGAAATCGCAGCA
ldhL (LSA1606)	ldhL-F	AGATATCGCTGAAATGGTTAACGTT
	ldhL-R	CATGTGACCATACAGGGAATTCTG
	ldhL-Taq	CGCACGTTCGGTCCATGCTTACATTA
gpm3 (LSA0206)	gpm3-F	TTACTTTGGAACGTGTTATCCCATT
	gpm3-R	GGCCGTCGATTAATTTTGGA
	gpm3-Taq	TGGGAAGACGAAAT

[a] Taq probes, 6-FAM, 6-carboxyfluorescein (fluorophore); TAMRA, 6-carboxytetramethylrhodamine (quencher).

Table S4. Comparison of microarray data with qRT-PCR results of *L. sakei* strain LS 25 grown on ribose compared with glucose.

Gene locus	Gene	Microarray $\log_2$ ratio[a]	qRT-PCR $\log_2$ ratio[a]
LSA0254	*lsa0254*	1.80	1.98
LSA0449	*manL*	1.53	1.79
LSA1606	*ldhL*	-1.46	-1.30
LSA0206	*gpm3*	-0.86	-1.17

[a] Gene regulation values ($\log_2$) are average results from three biological replicates.
